# Indoor environmental quality in schools: NOTECH solution vs. standard solution

**DOI:** 10.12688/f1000research.130633.1

**Published:** 2023-05-30

**Authors:** Carlo Volf, Klaus Martiny, Mathias Andersen, Bodil Engberg Pallesen

**Affiliations:** 1 NID-Group, Copenhagen University Hospital, Copenhagen, Frederiksberg, 2000, Denmark; 2 DTI, Danish Technological Institute, Aarhus, 8000, Denmark

**Keywords:** Natural ventilation – indoor environmental quality (IEQ) – schools – mechanical ventilation – energy performance

## Abstract

Background

In many Danish schools, the indoor environmental quality (IEQ) is challenging and studies document a poor IEQ in a majority of existing schools. Municipalities cannot afford comprehensive renovations and expensive mechanical ventilation solutions, hence public schools often suffer from poor indoor environment conditions. This study tests a new façade based, demand-controlled ventilation solution called NOTECH in the renovation of school. The study tests NOTECH vs. existing mechanical ventilation solution, comparing performance of both solutions at Skovbrynet Skole in Denmark.

Methods

The project implements and investigates the effect of the NOTECH solution in a primary school classroom, comparing it to a similar classroom with conventional, mechanical ventilation. Methodically, indoor environmental quality and energy performance is monitored in the two identical classrooms during one school year 2018 - 2019.

Results

The results show that both systems keep the conditions within acceptable limits and CO _2_ levels below 1000 ppm, which is the requirement according to the Danish Building Regulations. In terms of costs, the NOTECH system has a lower overall cost than the mechanical ventilation system, with total estimated costs for installation, heating, electricity and maintenance amounting to approximately 35% of the mechanical system’s costs. Finally, the results show that the NOTECH solution has a smaller embedded CO _2_ footprint for building materials, reducing the estimated carbon load by 95% compared to the mechanical ventilation solution.

Conclusions

The performance of the two systems roughly is the same in relation to the indoor environmental quality, temperature, CO _2_ levels, humidity and noise level. Costs for implementation, energy consumption for heating and CO _2_ footprint for building materials however, are significantly lower for the NOTECH solution, compared to the mechanical solution.

## Introduction

When it comes to establishing and maintaining a good indoor climate, schools have always been of particular interest. Historically, a good, balanced indoor climate was established in a very low-tech manner; pupils typically spending 45 minutes in each lesson, in high-ceilinged classrooms, often followed by 15 minutes of venting during breaks, in empty classrooms and with children outdoor in the school yard year-round. This meant that in most cases, natural ventilation - which most schools were fully based on until the mid1960s – probably did meet the requirement of CO _2_ < 1.000 ppm, based on natural air-intake from windows and doors, ^1^ the buildings at that time not being air-tight as today. ^2^^,^^3^ However, the situation and teaching philosophy is different today. First of all, schoolchildren spend more time inside the buildings. According to a report from Organization for Economic Cooperation and Development, OECD (2016), ^4^ Danish pupils spend a total of 10,960 hours on school education, which is considerably more time than the European average of 7,540 hours. Secondly, lessons tend to have a longer duration today, the children often having 2-period lessons corresponding to 1.5 hours. Thirdly, the architecture of the schools has changed. Together with energy renovation and air-tight mechanically ventilated buildings, new types of classrooms have been introduced, such as project space schools and open-plan schools. Older schools however, built before 1995, still have traditional classrooms based on natural ventilation through the windows. These schools make up 90% of all schools in Denmark and often suffer from a poor indoor environmental quality. A Danish study from 2016 by Toftum and Wargocki ^5^ showed that more than half of the existing schools in Denmark (1,300) suffer from a poor indoor air quality. Only 40-44% of schools in Denmark had a “good” indoor air quality (CO _2_ below 1.000 ppm).

A few years later, a study with 234 Danish schools and 709 classrooms found that 53% of the classes had higher CO2-concentration levels than the allowed threshold of 1000 ppm (Toftum, Clausen and Christensen, 2022) (25). This study showed largely the same result as the earlier study from 2016, despite a number of initiatives to correct the inadequate ventilation. Even though slightly more classes than before had a ventilation system, the air quality did not improve significantly. Based on measurements in 60 Danish schools, with a total of 245 classrooms, the researchers found that ventilation had a positive effect on sick leave among teachers. The study also showed that generally increased air change per hour resulted in students’ performance improving by9%. Furthermore, lowering the temperature from 25 °C to 21 °C resulted in an 8% improvement in student performance. However, 91% of 245 classrooms in Denmark exceeded the recommended upper limit of 1.000 ppm CO _2_ in some periods during the school hours. On average, the 1.000 ppm CO2 limit was exceeded during 47% of the school hours during the heating season (November – April) and 12% during the summer season (May - October). The results also indicated that general noise levels were too high (above 65 dB(A)) in the classrooms in 63% of the usage time.

These findings are supported by other studies by e.g. Heschong Mahone Group (1999), ^6^ Tanner (2009) ^7^ and Shendell *et al*., 2004, ^8^ which suggested that a poor indoor climate often resulted in more noise in the classroom, reduced concentration ability and poorer learning. Better air quality and better light quality also have shown to increase students' concentration and performance in mathematics and reading, among other subjects (Barrett *et al.,* 2015 ^9^ and Grün and Susanne Urlaub, 2015). ^22^

A study (Kjeldsen *et al.* 2015) ^10^ suggested a link between indoor climate parameters, such as room temperature (°C), CO _2_ levels (ppm), daylight (lux), relative humidity (%) and noise level (dB(A)), and performance, with these parameters having an effect on well-being, learning and general health of students. Economically, a Danish study in 2018 ^11^ showed that maintaining a good indoor climate costs approximately 0.38-0.54 EUR per pupil per day, using conventional, mechanical ventilation, corresponding to approximately 2.100 – 3.000 EUR per class annually. This represents a heavy burden for schools and municipalities, and may be the reason why the renovation of schools is happening slowly in Denmark. Approximately 50% of Danish schools were built in the 1960s and 1970s, and so many schools need renovations. Only approximately 10% of all schools have been built according to the energy requirements in the Building Regulations 1995 and onwards, and only a fraction of these, approximately 5%, are built according to the energy requirements for buildings from 2006. ^10^ In other words, more than half of the Danish schools face an urgent need for energy renovation and improvement of the indoor climate in order to meet the current requirements.

## Methods

Methodically, the study monitored the performance of the NOTECH solution and compared it to a traditional mechanical ventilation (MechVent) solution. The systems were implemented in identical, east-facing classrooms at Skovbrynet Skole in Copenhagen. The school was built in 1968 and chosen as it was representative of typical Danish schools, which mostly were built in the 1960s. These schools were originally based on natural ventilation through façade windows, but today they are often being renovated to full mechanical ventilation. Inclusion criteria for the class were set to 4 ^th^ – 5 ^th^ grade pupils representing the median for a normal 9-10 years period of schooling. The NOTECH system included façade elements, windows, blinded windows, solar chimneys for supplementary air outlet, and louvres for air intake with eelgrass filtering of outdoor air. Intelligent demand-controlled outlets on the top of the classroom and intakes at the bottom of the classroom were continuously adjusting the NOTECH ventilation rates according to temperature and CO _2_ levels inside and weather conditions outside; see Figure 2. In comparison, the mechanical ventilation system included conventional air-tight construction and conventional materials such as mineral wool and wood. The mechanical ventilation system was controlled by a clock timer in the intelligent building system (IBS).

**Figure 1.**
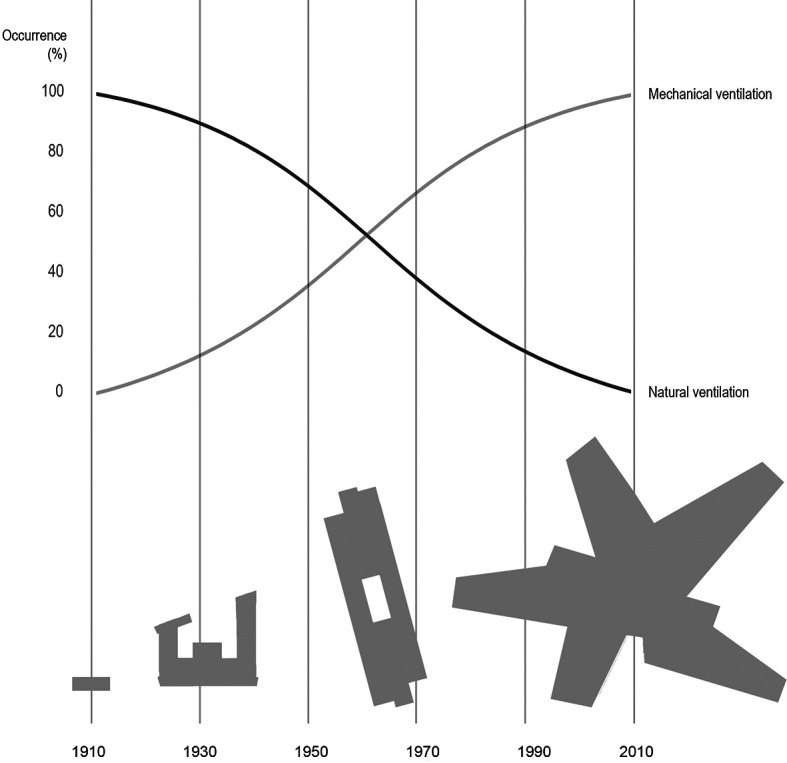
Illustrating a simplified development in typical Danish school typologies over the last 100 years in the period 1910 to 2010, and an estimated corresponding development in ventilation principles (top), changing from fully natural to fully mechanical ventilation. From the village school (1910) to the aula school and an early example of the open plan school, up to the project school.

**Figure 2.**
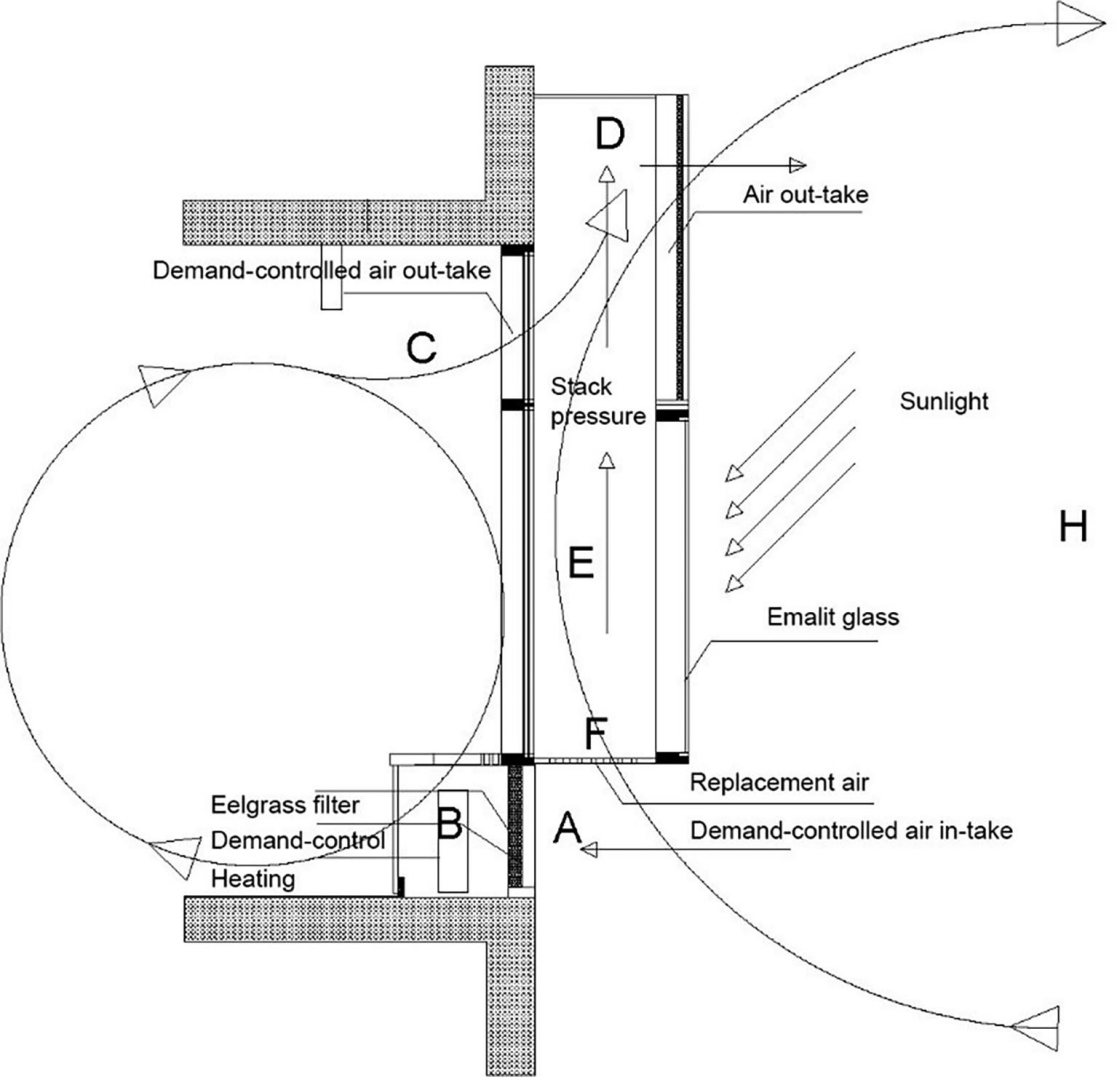
The NOTECH system, vertical cross section of façade. Top: A solar chimney (E, D) provides under-pressure and IoT demand-controlled dampers (A) control fresh air-intake at the same time filtering the fresh air and reducing outside noise through special designed eelgrass filters (B). Bottom: Each classroom has two solar chimneys outside with air outlet above ceiling height.

### Description of classrooms

The study was performed at Skovbrynet Skole. The public school was built in 1967 and renovated in 2002. Two east-facing classrooms were included in the study, both dimensioned for 24-28 pupils, each having a total of 16 pupils and 1-2 teachers. Both classrooms measured 9.5 x 8.6 m, with a net area of 81 m ^2^. The classroom with the mechanical ventilation system had a recessed ceiling with a ceiling height of 2.7 m. The classroom with the NOTECH system had a total ceiling height of 3.2 m, with no recessed ceiling, since NOTECH was a façade-based ventilation system. Thus, the volumes were 220 m ^3^ for the classroom with the mechanical system and 262 m ^3^ for the test room with the NOTECH system.

The two classrooms had identical materials, structure and furniture, except for the façades. In the classroom with the NOTECH system, novel natural insulating materials (eelgrass) and clear glazing (g = 0.72) were introduced, while the reference classroom was based on conventional materials (mineral wool) and standard glazing (g = 0.60). The total glass-area of the classroom with the NOTECH solution was 14 m ^2^ (glass-floor area ratio of 17.3%) whereas the total glass area of the MechVent solution was 16.1 m2 (glass-floor area ratio of 19.9%). Both classrooms had exterior yellow screens for solar protection and two openable windows.

### Description of the systems

The ventilation principles of the two classrooms were fundamentally different. The NOTECH system in the test classroom was a passive ventilation system, driven by thermal stack pressure, while the system in the reference classroom was driven by a central, mechanical ventilation unit. Both systems fulfilled the minimum requirements of 5 l/s/pupil and 0.35 l/s/m ^2^ floor area. The ventilation in the reference classroom was similar to that of the rest of the school.

The MechVent solution

The existing mechanical ventilation system was based on a standard, central unit serving a number of other classrooms (28), driven by motors running at an installed power of 2x4 kilo-Watts and demand-controlled by a clock timer in the IBS system. Via a (IBS) timer, the system was set automatically to start ventilating the classroom at 7 am, one hour before the start of teaching and end at 5 pm. The capacity of the standard mechanical ventilation system was dimensioned for a maximum airflow of approximately 500 m ^3^/h per classroom. During the experiment, the airflow was estimated to be running at 80% of the full capacity.

*The NOTECH solution*

The capacity of the NOTECH system was dimensioned to a maximum airflow of approximately 750 m ^3^/h per classroom. The demand-control of the NOTECH system was controlled by being tuned to an estimated airflow of 50% of full capacity, based on the number of pupils. The NOTECH system was set to automatically ventilate from 07.30 am and end at 3 pm, since the demand-controlled system only ventilated during the usage hours. The tuning was regulated twice, on October 6 ^th^ 2019 from 20 to 22°C and again the Dec 20 ^th^ to 21.5°C. The later adjustments were based on a wish to optimize and balance the ventilation rate, at the same time making sure that no pupils nor teachers found the room too chilly. Adjustments only affected the setpoint of temperature, not the setpoint of timer.

The total outlet area of NOTECH was 0.7 m ^2^ and had a maximum air velocity of approximately 0.3 m/s. The air intake was 2.1 m ^2^, approximately three times larger than the outlet area, allowing lower airflow speed at high air change per hour (ACH), without the risk of draught. At the same time the NOTECH system was designed to reduce noise, using eelgrass as a noise-reducing, acoustic material. The NOTECH system also included two supplementary solar chimneys, enabling potentially higher ventilation rates during sunny days. The solar chimneys may potentially have an effect, especially on the summer ventilation, causing more passive cooling/ventilation during sunny days ( Figure 2). This effect was not studied in this project.

The NOTECH system integrated three elements 1) natural ventilation 2) clear glazing and 3) natural materials, as described below.

*Natural ventilation*

NOTECH was based on passive, one-sided thermal ventilation; taking advantage of the solar heat radiation as a supplementing force to enhance the buoyancy effect. The solar chimney ventilation system was designed to reduce excessive temperatures in the classroom during summer periods. In most cases this system means that the original building physics can be maintained, so that recessed ceiling heights may be avoided and only windows replaced.

*Clear glazing*

The system introduced high-transmittance windows based on two-layered low-iron clear glass that provided more transmitted daylight in the form of UV, visible and IR light. ^12^ The window allowed a clearer view out. It also allowed parts of UVB light to penetrate, which may have positive health effects such as enabling vitamin D formation, ^13^ as well as germicidal effects and killing of bacteria/viruses. ^14^ During winter periods, the NOTECH solution was designed to utilize to a higher degree solar light and heat, using high transmitting, clear glazing, with a higher g-value, thus facilitating a higher passive solar heat gain in the heating season.

*Natural materials*

The system introduced traditional, vernacular materials such as eelgrass combined with natural ventilation using intelligent demand-control technology. Natural materials such as eelgrass are known to have a antimicrobial effects, because of its natural salt-content and antimicrobial secondary metabolites (Choi *et al.*, 2009; Papazian *et al.*, 2019) (22, 24). Traditionally, eelgrass was applied in mattresses and indoor furnishings, etc. When applied in larger quantities, these natural materials help maintain a higher, more natural, relative humidity, being a hygroscopic building material, absorbing and releasing moist. ^15^ In this way, eelgrass helps balance the humidity, maintaining a relative humidity above 40% RH, which is a recommended minimum threshold value for a healthy environment. ^16^^,^^17^

*Monitoring procedure and time plan*

Data were collected continuously over one year and analyzed for representative 3-week periods during the summer and winter, respectively. Typical periods were chosen as 3-week periods with temperatures above 20 C during summer and 3-week periods with temperatures below 10 C during winter. This was in order represent a normal Danish summer and winter setting. Data were simultaneously collected for both classrooms (NOTECH and standard mechanical ventilation) using two identical Indoor Climate meters (IC-Meter Mobile (GSM), in each classroom, placed 1.8 m above the floor in each classroom. IC meters were placed at identical locations, in the center of both inner walls in the two classrooms. Indoor climate parameters CO _2_, temperature, relative humidity, and noise were measured and logged every 5 minutes. Data were extracted for representative weeks for the summer period and the winter period, week 35/37 in 2019 (26.08.19 – 15.09.19) and week 8/10 17.02.20 – 11.03.20) in 2020, respectively; these periods being the warmest and coldest periods. See Table 1.

**Table 1.** Time plan 2019-2020 showing baseline measurements and effect measurements. Baseline measures were not included in the results and only served as a baseline for the parallel health study to confirm that the two classrooms were identical before intervention.

PERIOD	Baseline	Measurement dates	Representative weeks
Summer period (off heating season)	08.03.19. – 15.03.19	15.08.19 – 21.10.19	26.08.19 – 15.09.19
Winter period (heating season)		22.10.20 – 11.03.20	17.02.20 – 11.03.20

### Description of data collection

*Indoor environmental quality*

Hourly average values of CO _2_ concentration, relative humidity, noise and temperature were analyzed for the representative weeks, as averages, cumulated frequency of different CO _2_ levels, relative humidity (% RH), temperature and noise levels. Data on the two glass types used in the study document a higher transmittance of the NOTECH system’s glazing types (g = 0.72, visible light transmittance = 0.82) providing more transmitted daylight and solar heat compared to the mechanical system (g = 0.6, visible light transmittance = 0.74). Differences in daylight between the classrooms were not measured, since the focus of the study was on CO _2_ levels, relative humidity (% RH), temperature and noise levels.

*Energy performance*

The operation costs for electricity of both systems, excluding energy costs for lighting, were calculated as EUR per pupil per month in total estimated electricity consumption, based on a Danish national kWh price of 0.30 EUR (2019/2020), with an estimated average of 20 children in each classroom, 7 h per day, and the standard usage period of 25.2 days per month (corresponding to 200 days per year).

Operation costs of both systems were based on measurements using digital Ista Oprimo III evaporation meters. The evaporation meters were installed directly in front of the radiators in each classroom 0.5 m above floor level. Installation was carried out at the same day and evaporation meters were monitored every 15 minutes during the heating season.

*Total installation costs*

In order to represent a realistic school setting, the calculations of energy performance for electricity and heating, together with the estimated installation costs, were based on a total of 10 classrooms for both systems. All operating costs for electricity, heating and maintenance together with installation costs were based on a 20-year lifetime, which is considered the lifetime of conventional, mechanical ventilation systems.

*Carbon footprint*

The approximated life-cycle analysis (LCA) was based on the European ECO2 carbon footprint for materials and products with a focus on wooden building products. ^17^ Material calculation for the mechanical system was based on data and description of materials from the suppliers. Material calculation for the mechanical system was based on one central, mechanical system, Danvent Combi Aggregate TC, with heat recovery running on 240 V, with galvanized steel ducts. Material calculation for the NOTECH system was based on 2 pcs IoT-controlled chain actuators (WMX 804) in steel and 4 pcs Klimatek dampers in aluminum, running on 24 V Belimo motors.

*Parameters for intelligent demand-control*

The NOTECH system was demand-controlled by three parameters: usage time, temperature levels and CO _2_ levels, taking into account the number of pupils. The standard mechanical system was controlled by one parameter: usage time, not taking into account the number of pupils in the classroom.

*Analysis*

Indoor environmental quality parameters for a 3-week representative period during summer and winter, were represented in grouped in intervals in pie charts showing a percentage of the different ranges observed in order to compare the two systems across seasons.

## Results

### Indoor environmental quality

The overall results of the indoor environmental quality (Volf, 2023) showed the following differences between the two systems.

**Figure 3.**
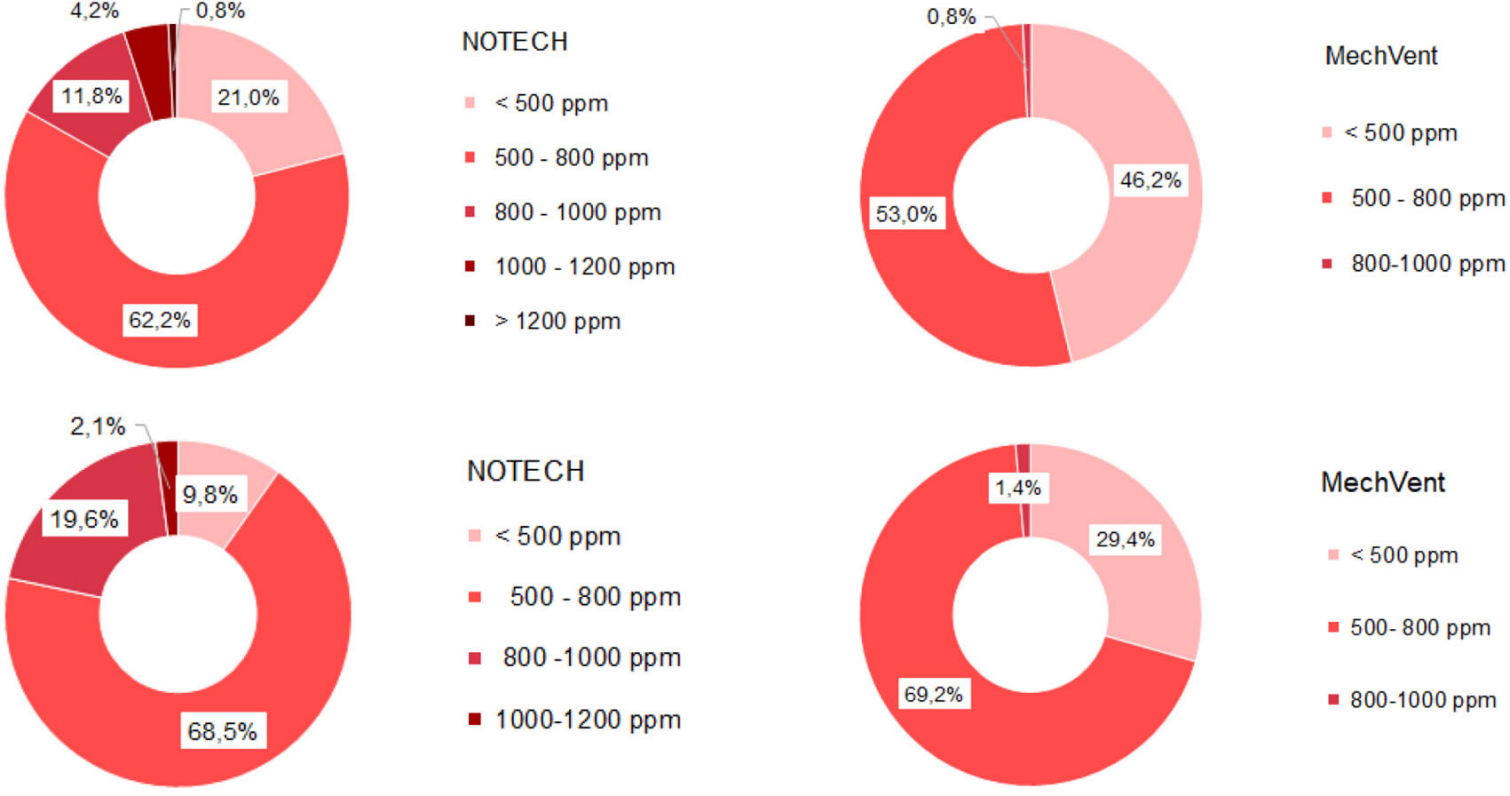
Frequency (%) of indoor classroom CO _2_ levels (without correction for outdoor CO _2_ levels). NOTECH and mechanical ventilation summer (above) and winter (below)

**Figure 4.**
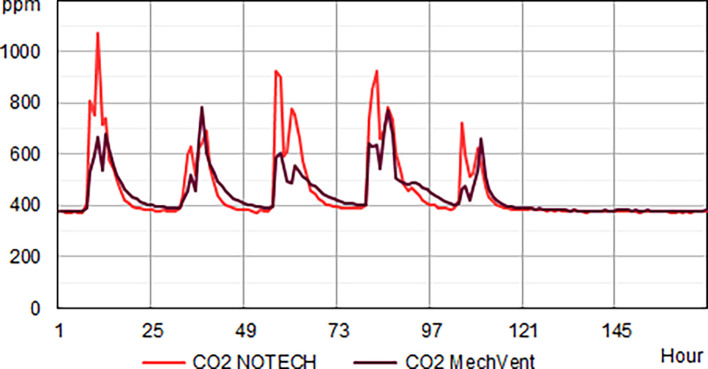
CO _2_ levels for one winter week. Typical average hourly levels for NOTECH natural ventilation compared to mechanical ventilation (MechVent).

Indoor classroom CO _2_ levels were lower than 1.000 ppm for 100 % of school hours for the mechanical system and 95% of the school hours for the NOTECH system in the summer period 2019 and winter period 2020 ( Figure 3). CO _2_ levels above 1.000 ppm were reached for the NOTECH system in short periods of 10-15 minutes, 5 % of the usage time, and only during the summer period ( Figure 3). CO _2_ levels for the mechanical system generally were lower than for the NOTECH system ( Figure 4). On average during the summer and winter period, the CO _2_ levels were 600 ppm for the mechanical vs. 800 ppm for the NOTECH system. Higher CO _2_ levels were reached during the summer period than during the winter period for the NOTECH.

**Figure 5.**
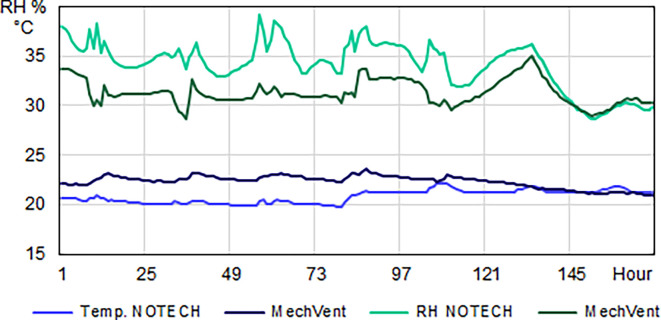
Temperature (°C) and relative humidity (%) for one winter week. Typical hourly average levels for NOTECH natural ventilation compared to mechanical ventilation (MechVent). Because NOTECH uses cold “fresh” outside air, the indoor temperature is lower (20 °C) and the relative humidity (%) higher compared to mechanical ventilation.

**Figure 6.**
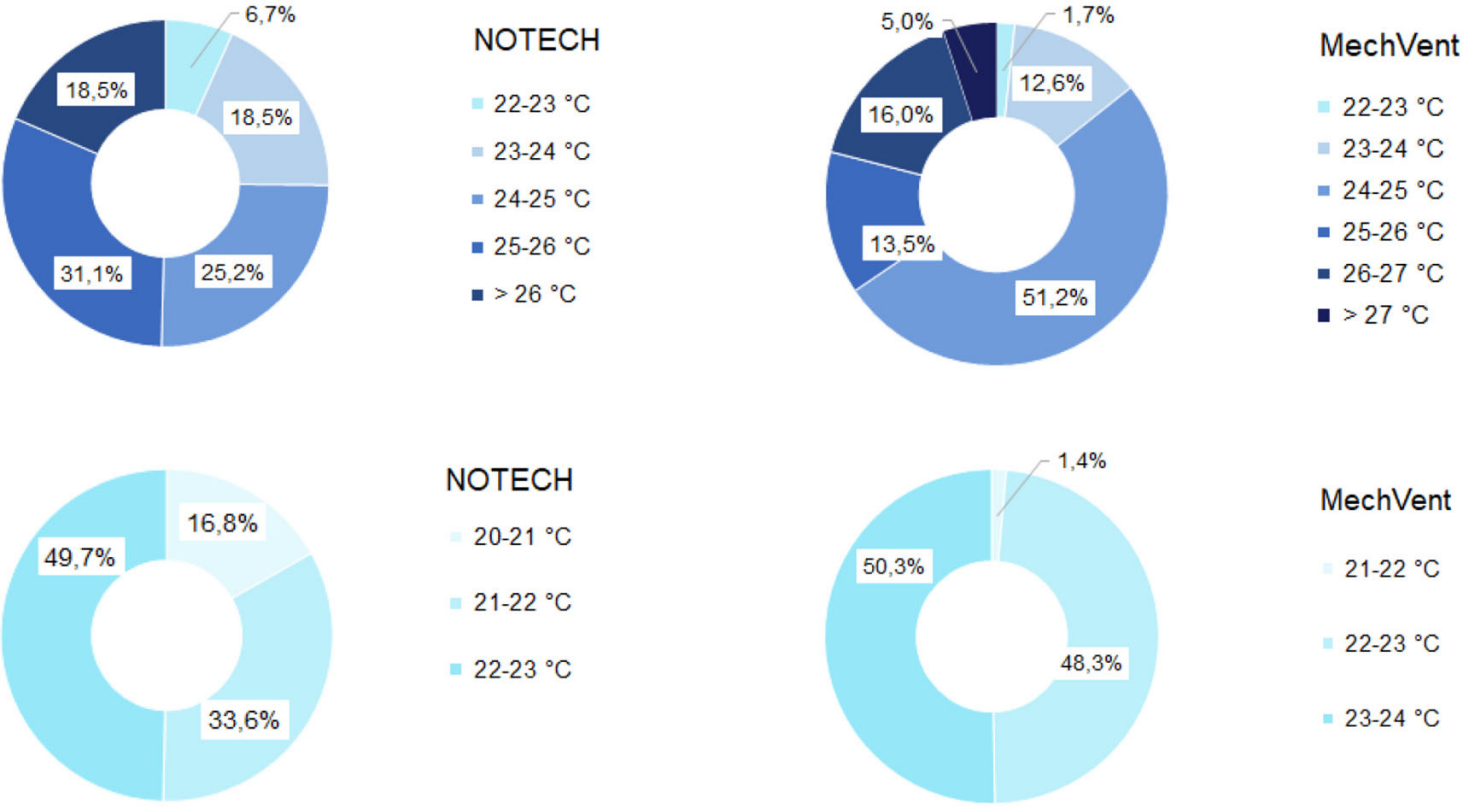
Frequency of temperature (% **)** for NOTECH and mechanical ventilation in summer (above) and winter (below).

NOTECH uses outside air directly without preheating, hence control of the indoor temperature (> 20°C) was a determining factor for the ventilation rates. MechVent, using preheated air, had higher air rates, which reduced the CO _2_ levels compared to natural ventilation. The indoor temperature for the NOTECH system was typically about 2-2.5 °C lower than the temperature for the mechanical system (MechVent) ( Figure 5). For the NOTECH system, comfortable temperatures of 20-24°C were obtained 25.2% of the time in the summer period, with no hours with temperatures above 27°C. For the mechanical system, comfort temperatures of 20-24°C were obtained 15.3% of the time in summer, and the hours with temperatures above 27°C added up to 5% of the usage time ( Figure 6).

The high-transmittance glazing types (g = 0.72) in the NOTECH system did not cause more hours with temperatures above 27°C during the summer period. Temperatures above 26°C were observed 18.5% of the time for the NOTECH system, compared to 21% of the time for the mechanical system, Figure 6.

Temperatures during winter were generally colder for the NOTECH system, with the lowest temperatures recorded between 20 and 21°C, observed 16.8 % of the usage time, whereas no temperatures below 21°C were observed in the classroom with the mechanical system.

**Figure 7.**
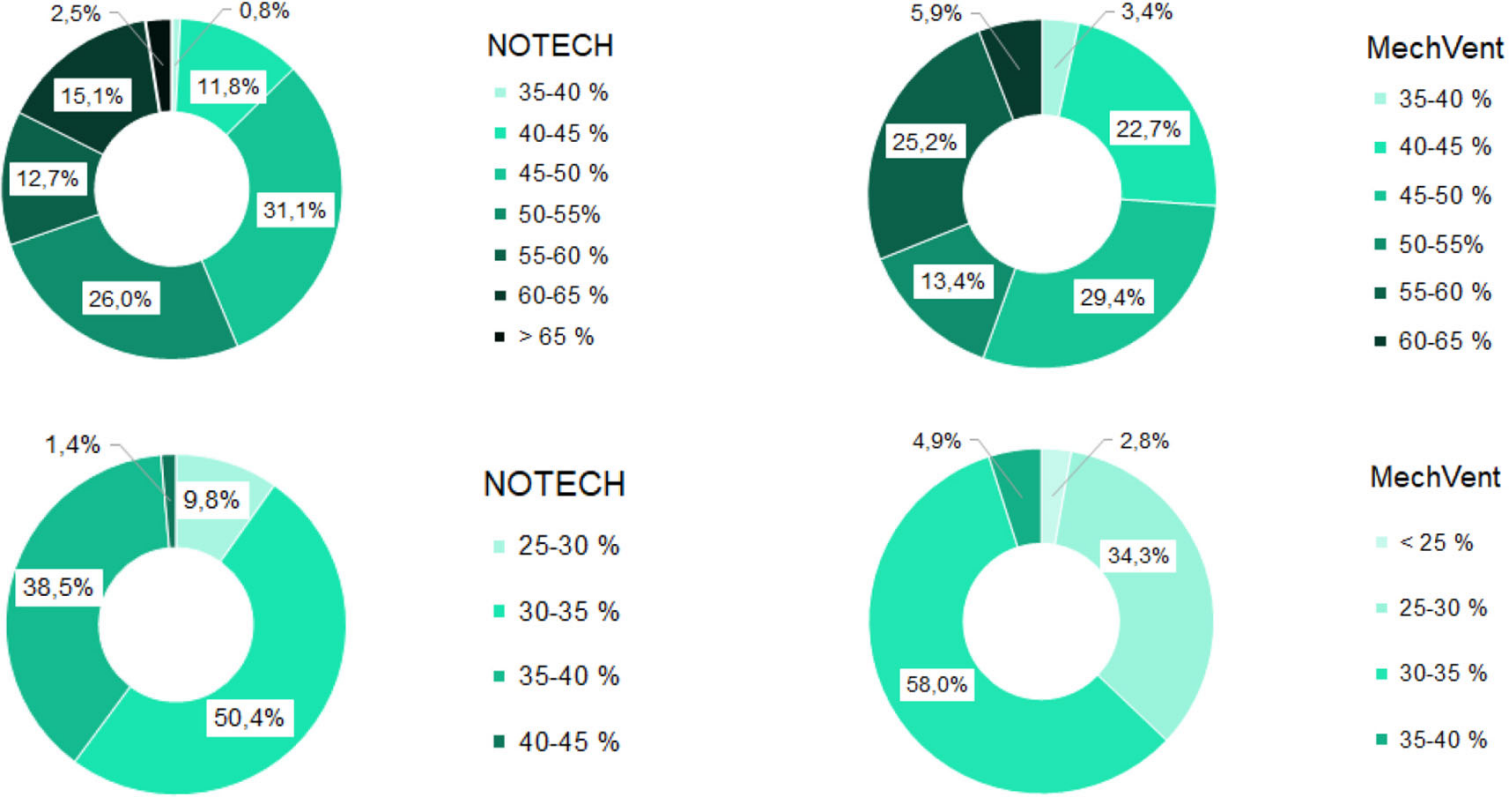
Frequency (%) of relative humidity For NOTECH and mechanical ventilation in summer (above) and winter (below).

During the summer period, the relative humidity was roughly the same for both systems, see Figure 7. This is despite the fact that the temperatures generally were higher for the classroom with the mechanical system. During the winter period, the relative humidity for the NOTECH system was between 35-45% RH 39.9% of the time, while a relative humidity between 35-45% was observed only 4.9% of the time for the mechanical system. The general indoor winter temperatures were however higher for the mechanical system during winter, thus lowering the relative humidity; see Figure 6.

**Figure 8.**
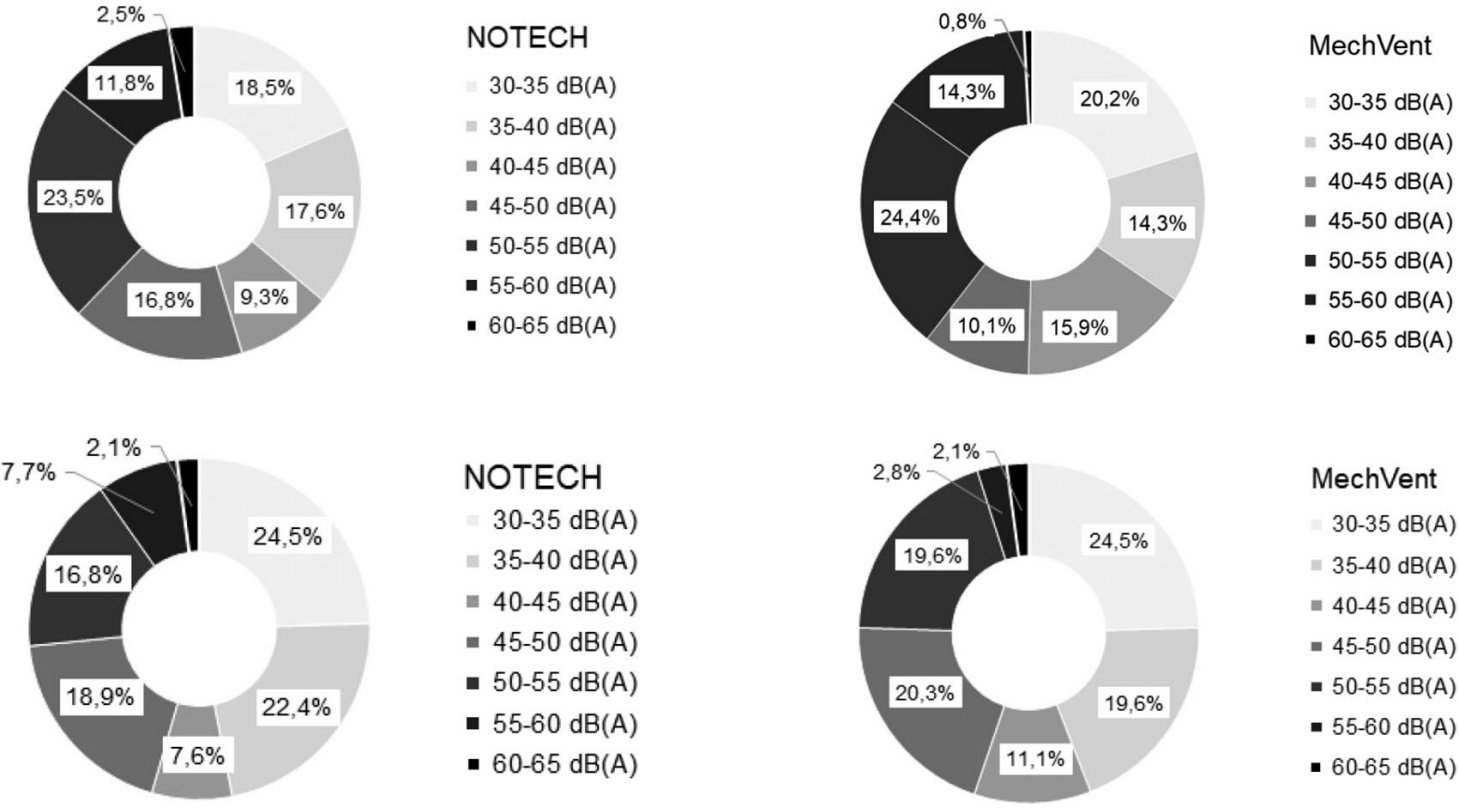
Frequency (%) of noise (dB(A)) for NOTECH and mechanical ventilation in summer (above) and winter (below).

Noise levels dB(A) during the school hours showed no measurable difference between the two systems. Noise levels above 50 dB(A) are more frequent during the summer period for both systems (37.8% NOTECH system - 39.5% MechVent). Noise levels above 50 dB(A) are less frequent during the winter period (26.6% NOTECH system - 24.5% mechanical system), Figure 8.

Average daylight levels indoors were not measurably different between the systems, although more daylight was transmitted through the two layered low iron glazing of the NOTECH solution compared to that of the standard glazing in the room with the mechanical system. The glass area, on the other hand, was reduced in the NOTECH system.

### Energy cost

The energy costs of electricity for both systems were estimated based on a total of 10 classrooms with 20 pupils 7 h per day, 25.2 days per month and kWh price set at 0.30 EUR. The estimated annual running cost for electricity was based on a 20 year lifetime. Estimated annual electricity consumption per classroom for the standard mechanical system was 305 kWh, amounting to 91 EUR annually per classroom. Estimated annual electricity consumption per classroom for the NOTECH system was 7 kWh, amounting to 2 EUR annually per classroom.

**Figure 9.**
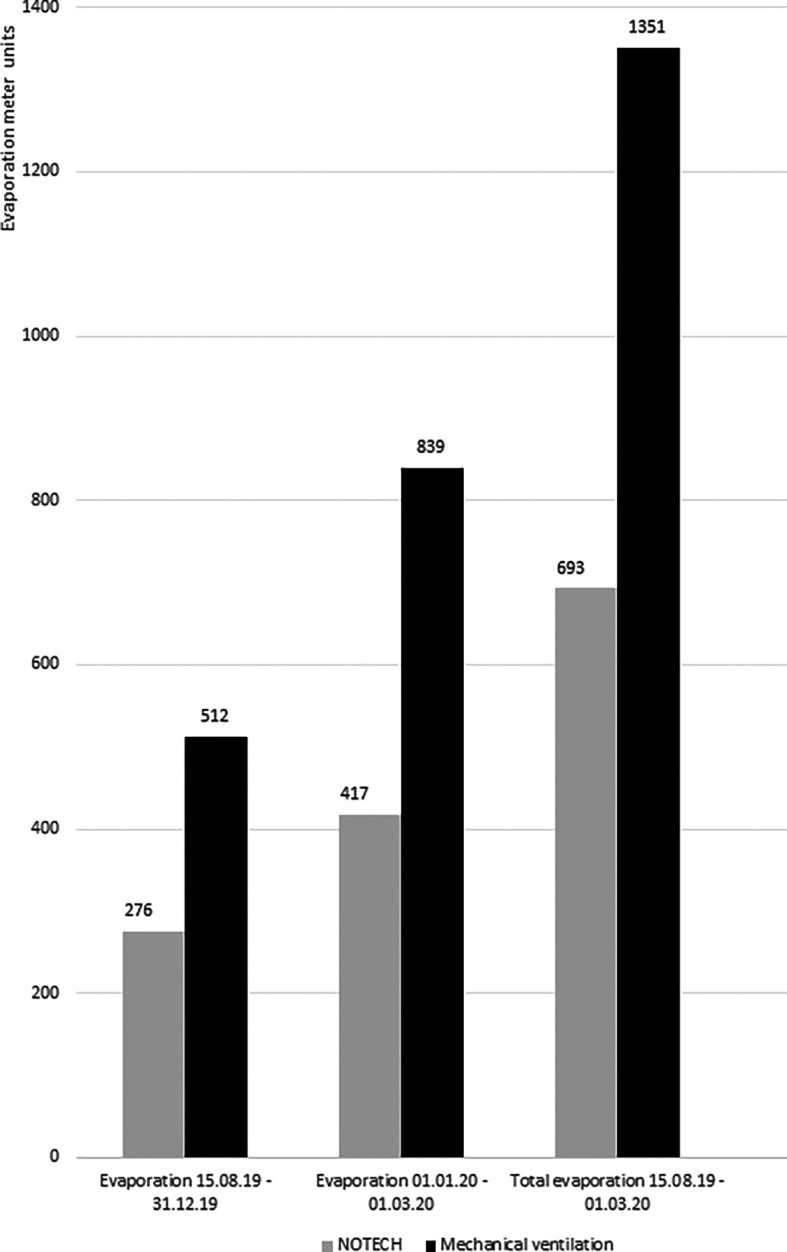
Evaporation meter registrations for NOTECH system vs. mechanical ventilation. The monitored data from evaporation meters monitoring each system during two periods. The first period is 15.08.2019-31.12.2019 and second period is 01.01.2020-01.03.2020. Source: Ista-meter 839, 891, 952, 072.

Energy consumption for heating was measured in the classrooms for NOTECH and the mechanical system, respectively. The energy for heating during the heating season (October-March), as measured by Ista evaporation meters, showed cumulated 693 units for the NOTECH system and 1,351 units for the mechanical system. Monitoring was carried out in two periods, the first period was for 2019 (15.08.2019-31.12.2019) and second period was for 2020 (01.01.2020-01.03.2020) ( Figure 9).

Based on data from Gladsaxe Municipality, the degree day corrected total energy consumption for Skovbrynet Skole for heating October 2019 – April 2020 was 1,358 MWh for a gross heated area of 11,080 m ^2^, giving the following average energy consumption for the two systems based on 81 m ^2^ classrooms and 0.30 EUR per kWh. Based on Ista measurements, the energy consumption for the heating season 2019/2020 was 62,9 kWh/m2 per year for the NOTECH system and122,6 kWh/m2 per year for the mechanical system. Costs for heating per classroom in the heating season 2019/2020 amounted to1.540 EUR per year for the NOTECH system and 3.000 EUR per year for the mechanical system, based on 0.3 EUR/kWh.

### Installation cost

The estimated total installation costs were calculated based on data from Skovbrynet Skole, based on information from suppliers of the NOTECH system (WindowMaster Ltd and Velfac Ltd) and the mechanical system (DanVent Ltd). All installation costs were based on 10 classrooms, each having 20 children for both systems and a lifespan of 20 years. The total installation costs for the NOTECH system were all in all 48.450 EUR and the total installation costs for the mechanical system were 147.800 EUR (40.300 EUR for the ventilation system and 107.500 EUR for ducts, fixtures, filters, etc.).

### Total estimated costs

The total estimated costs for energy, installation, heating and maintenance per day per pupil (based on 20 pupils per classroom and 200 school days per year) was 1.06 EUR for the NOTECH system and 3.00 EUR for the conventional mechanical system. All in all, the total annual estimated costs of the NOTECH system amounted to 35% of the conventional, mechanical system; see Table 2.

**Table 2.** Total annual estimated costs for energy, installation, heating and maintenance (every half year based on an estimated, average 20 year lifetime).

	Electricity	Heating	Installation	Maintenance	Total
	EUR/classroom per year	EUR/classroom per year	EUR per year	EUR/classroom per year	EUR/classroom per year
NOTECH	2	1.540	2.400	300	4.242
MechVent	91	3.000	7.650	1,250	11.991

### CO _2_ in building materials

**Figure 10.**
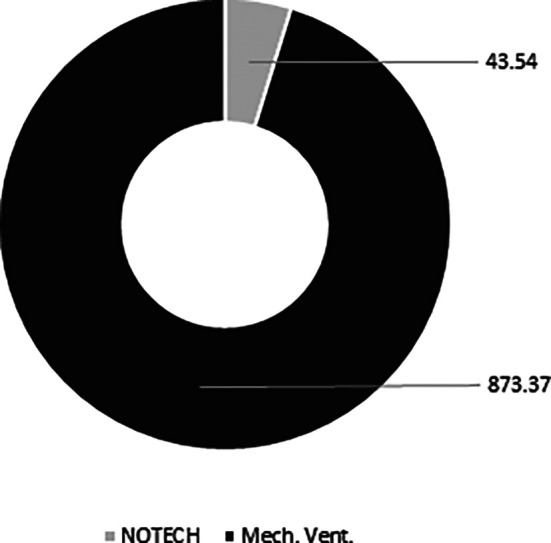
Estimated overall carbon footprint (kg CO2) for NOTECH system vs. mechanical system based on 10 classrooms. Embedded CO _2_ in the building materials is estimated for a standard life time period of 30 years. ECO2 data for materials used is standard values from Ruuska. ^18^

The NOTECH solution was based on natural, bio-based materials, thus the CO _2_ footprint was smaller. The conventional mechanical solution spent 873 kg CO _2_, while the NOTECH solution spent 44 kg CO _2_. Thus, the NOTECH system reduced the embedded CO _2_ in the building materials by a total of 95%, compared to the mechanical ventilation system ( Figure 10).

## Discussion

The aim of this study was to make a comparison between the NOTECH system and a conventional mechanical ventilation system. The classrooms in the study were representative for schools from the 1960s and 1970s and the results of this pilot study therefore should be seen as “a proof of concept” for this type of schools. The two rooms were alike though differing in volume due to the recessed ceiling height (0.5 m) in the classroom with the mechanical ventilation system – a difference which is considered representative for the solutions, since NOTECH as a façade solution, is not taking up ceiling space. The usage of the rooms were comparable, each hosting 16 fourth/fifth grade pupils.

### Indoor environmental quality

The airflow in the mechanical system was controlled by a clock-timer, while the NOTECH system was demand-controlled by three parameters: usage time, temperature levels and CO2 levels. Except for short periods, both systems could keep the CO _2_ levels and temperatures within acceptable limits under the given conditions in the actual measurement periods.

CO _2_ levels

Generally, higher values of CO2, temperature, humidity and noise tended to follow a higher activity and person load in the classrooms. Lower CO _2_ levels were generally observed during the summer period, compared to higher levels in the winter period. For both systems the summer period showed increased hours with low CO _2_ levels (below 500 ppm), indicating that the pupils were spending more time outdoor during summer – since inside activity at these periods would have shown an increase in the CO _2_ levels (above 500 ppm). However, higher CO _2_ levels were reached during the summer in the NOTECH solution. The natural, temperature gradient driven ventilation in NOTECH did not provide a constant air change per hour. The demand-control of the NOTECH system was tuned and slightly adjusted during the winter period, to provide a slightly higher air change during the winter period. We cannot tell if these adjustments caused lower CO _2_ levels in general during winter. Windows could be manually opened in both classrooms and other unknown factors were present.

Normally the winter period is “the critical” period for natural ventilation systems, however, the demand-control of the NOTECH system in this pilot study seems to meet the requirements, providing sufficient fresh air in 95% of the usage time at a lower cost, during both the summer and winter periods being tested. However, it should be kept in mind that the winter 2019/2020 was mild compared to a normal Danish winter.

Temperature

The difference in spatial volume between the systems is typical and representative for the two different systems. However, ceiling heights and room volumes may vary from building to building, and today´s mechanical systems may even have lower recessed ceilings. Increased ceiling height acts as a buffer reducing excessive temperatures. This may be another explanation for the higher frequency of temperatures above 26 °C in the classroom with the mechanical system, compared to the classroom with the NOTECH system.

The results show a lower number of hours with temperature above 26°C for the NOTECH system during the summer period, compared to the mechanical system (18.5% for NOTECH compared to 21% for mechanical ventilation) ( Figure 6). The fact that the glass area in the NOTECH system was reduced by 13% compared to the standard mechanical system may explain these results. The higher frequency of hours with temperatures above 26 °C (21%) in the standard mechanical system may also be explained by the fact that the NOTECH system provides generally lower room temperatures, passively cooling during the morning and daytime. Difference in air volumes in the classrooms may also have influenced the temperatures. Increased ceiling height and thus greater air volume acts as a buffer, reducing excessive temperatures. Taken together, these factors may all add up to explain the differences between the two systems.

Relative humidity

During the winter period, the relative humidity generally tended to be higher for the NOTECH system. The lower limit <30% RH was reached more frequently for the mechanical system (37.1%) compared to the NOTECH system (9.8%) ( Figure 7). This was partly caused by the general lower temperature in the classroom with natural ventilation. The heat recovery of the mechanical ventilation system together with differences in airflow – the NOTECH system being laminar and the mechanical system being turbulent airflow – may have had an influence on this, but the effects were not examined in detail in this study. In addition to a single-sided focus on set limits of CO _2_ concentrations, these results may suggest to include other parameters for a more satisfying indoor environmental quality, e.g. a better relative humidity.

Noise levels

The measured average noise levels (dB(A)) were not significantly different between the systems. Generally, indoor noise levels exceeded outdoor noise levels. Levels above 50 dB (A) were more frequent during the summer period (37.8% of the usage time for the NOTECH system - 39.5% of the usage time for the mechanical system) and less frequent during the winter period (24.5% of the usage time for the mechanical system - 26.6% of the usage time for the NOTECH system).

### Energy

This study reveals several differences between the two systems when it comes to energy performance and sustainability. The NOTECH system in general provides a sufficient air-change-per-hour at a lower estimated total annual cost. Estimated annual costs are 65% cheaper for the NOTECH system than for the mechanical system.

This study found that NOTECH saved energy for heating: 59.7 kWh/m ^2^ per year compared to the standard system, corresponding to a reduction of 51% for heating. This was not expected, the NOTECH system being a passive system taking in cold, outside air directly without preheating. The energy consumption for heating is based on dynamic factors (such as solar heat gain, weather/wind/outdoor and indoor temperatures, etc.); the function of the NOTECH system needs to be investigated further, especially during cold winter periods. Generally, the NOTECH system had a lower air change per hour which may explain the lower energy consumption for heating compared to the mechanical system. The energy consumption for heating fluctuates from year to year and data in this study were collected empirically during one year and through a mild winter period. A cold winter period may result in colder indoor air-temperatures or in a higher general energy consumption for heating, and remains to be examined further during cold winter periods.

### Costs

Our estimated installation and running costs for the mechanically ventilated indoor climate system in this pilot study are in good agreement with a previous study. ^10^ Based on 10 classrooms and 20 years lifetime, the installation cost for the NOTECH system is estimated to save 66%, compared to the conventional, mechanical ventilation system. The difference between the two systems in the estimated total capital expenditure (operating costs for electricity, heating, maintenance and installation costs) shows that the NOTECH system has a markedly lower cost compared to the mechanical system, costing only 35% of a mechanical ventilation system in total running costs. Based on a classroom with 20 pupils, the estimated total costs for the NOTECH are 1.06 EUR per day per pupil, while the costs for the mechanical ventilation system are 3.00 EUR per day per pupil.

More than half of the Danish schools face a need for energy renovation and improvement of the indoor climate in the form of optimized ventilation. In this respect, the results of this study show that systems such as the NOTECH can be an alternative to mechanical systems, resulting in fewer changes in existing architecture of the schools and less CO _2_ emissions and energy consumption.

With this background, the development of ventilation that merely relies on mechanical systems ( Figure 1) should be questioned. Mechanical systems are not the only solutions and supplemented by intelligent, natural ventilation systems, a balanced indoor environment may be obtained, at a far cheaper price than only relaying on mechanical systems. Today, existing schools often cannot afford expensive mechanical ventilation systems. Intelligent systems like NOTECH can be an alternative, avoiding technical retrofit changes in existing architecture, suspended ceilings and ducts, etc. A system such as the NOTECH system will often be technically easier to implement than a mechanical system, both when it comes to existing and new building constructions. This includes the fact that lowering existing ceiling heights can be avoided, which means that the system uses fewer materials, at the same time maintaining more space, both in the form of more volume (which means increased buffer against excessive temperatures) as well as more area due to the need of fewer and smaller technical rooms.

According to statistics in Denmark ^19^ approximately 710,000 students are attending grades 0 – 10 in public schools, based on a recent 5-year period 2015 – 2020. Assuming that 50% of these students attend schools build in the 1960s and 1970s, there is a market of approximately 355,000 students. Assuming NOTECH is distributed in classrooms, corresponding to 25% of these 355,000 pupils, with savings of 1.98 EUR per pupil per day, this corresponds to a total reduction in national costs of 33.145.000 EUR per year, compared to conventional, mechanical ventilation solutions.

### Life cycle assessment

The approximated LCA analysis, based on the European ECO2 carbon footprint for building data for materials and products with focus on wooden building products, showed that the NOTECH system had a far smaller CO _2_ footprint, being only 5% of the CO _2_ footprint of the mechanical system (see Figure 10). In this respect, NOTECH is better in line with future sustainability requirements such as e.g. Level(s) in the European Commission and Denmark’s New Class of Sustainable Architecture. ^20^ According to a recent study, ^21^ waste and materials from the building industry today make up 50-83% of the emitted CO _2_ over a period of 80 years. Making an effort to reduce the carbon footprint of the building materials by 95% is a significant and valuable contribution to a more sustainable architecture and an important result of this pilot study of the NOTECH system.

## Conclusion

This pilot study shows a proof-of-concept for the NOTECH system in typical school settings at Skovbrynet Skole built in 1967 and renovated in 2002. Further studies are needed to conclude that the demand-controlled system can also meet the acceptable levels under dimensioning conditions, i.e. with 24-28 students in the classrooms and during colder winter periods. The classrooms are representative for schools built in this period and the findings are only representative for similar cases. Likewise, the following conclusive points based on the specific case study are:

• With the person load of the classrooms and considering that the NOTECH system was controlled by CO _2_ in the indoor air, both systems could keep both the CO _2_ level and temperature within acceptable limits. CO _2_ levels were on average lower for the mechanical system, compared to the NOTECH system.
• The indoor room temperature was generally lower for the NOTECH system compared to the mechanical system. Having a lower set-point when compared to the mechanical system, the NOTECH system resulted in more hours with comfortable temperatures between 20-24°C compared to the mechanical system and fewer hours of temperatures above 27°C during the summer period when compared to the mechanical system.
• The relative humidity was higher for the NOTECH system, corresponding to a generally lower indoor room temperature. The lower limit <30% RH was reached more frequently for the mechanical system (37.1%) compared to the NOTECH system (9.8%). Variations in temperature and relative humidity generally tended to follow high activity and person load in the classrooms.
• Noise levels dB(A) measured during the school hours showed no measurable differences between the systems, indicating a similar activity level in the classrooms.
• The estimated total costs for energy costs (energy consumption for electricity and heating) were lower for the NOTECH system compared to the mechanical system. The energy consumption for heating showed that the NOTECH system accounted for only 51% of the energy consumption, corresponding to 62.9 kWh/m ^2^ per year compared to 122.6 kWh/m ^2^ per year for the mechanical system, during the heating season tested (October 2019 - April 2020). Usage time between the two systems was different. Usage time for NOTECH was 07.30 – 14.30 and 07.00 – 17.00 for the mechanical system, and this accounts for some of the energy excess energy consumption of the mechanical system. Also, the setpoint for heating was lower for the NOTECH system (20.5 C) compared to the mechanical system (22 C). The test period was a mild winter period and this affected the energy consumption, however this was the case for both systems.
• The total estimated installation costs for the NOTECH system amounts to 35% of the total installation costs of the conventional, mechanical system used in this study. However, the systems had a different start-time and end-time; NOTECH had a shorter operation time, 2.5 hours shorter than the mechanical system.
• Over a period of 20 years, the estimated total running costs of the NOTECH system were 65% cheaper than the estimated total running costs of the mechanical system.
• According to the life cycle assessment (LCA) over 30 years, the NOTECH system was estimated to reduce the carbon emissions of the building materials, the embedded CO _2_, by 95% compared to that of the mechanical ventilation system.

Since the NOTECH system is a passive system, primarily using energy for heating, the winter period is decisive for the total energy balance of NOTECH. In this study, the results show that NOTECH reduced the energy consumption for heating during a mild winter period – which is contrary to what could be expected during a normal Danish winter. This needs to be further investigated.

This pilot-study challenges the current building requirements when it comes to demands for mechanical ventilation. Today, it is mandatory to use mechanical ventilation systems with heat recovery, thus hindering systems such as NOTECH in the planning of school classrooms in Denmark. The results indicate that intelligent control of passive, natural ventilation systems can be a sustainable solution, if not fully replacing a mechanical ventilation system then supplementing mechanical systems. The NOTECH system did have lower energy consumption, less impact on the architecture of the school, used fewer materials and therefore had a lower environmental impact. The results suggest that other types of buildings, with rooms having a lower person load than classrooms, can also benefit from demand-controlled natural ventilation systems such as NOTECH.

## Ethical considerations

This study did not include any human participants.

## Data Availability

Zenodo: Indoor Environmental Quality in Schools: Notech Solution vs. Standard Solution. https://doi.org/10.5281/zenodo.7821202 (Volf, 2023). This project contains the following files:
- IC-Meter-QR-Indoor-Outdoor-Hour-Data-Aug-2019_MECH_VENT_5X.csv (mechanical ventilation data for summer 2019) - IC-Meter-QR-Indoor-Outdoor-Hour-Data-Aug-2019_NOTECH_5Y.csv (NOTECH ventilation data for summer 2019) - IC-Meter-QR-Indoor-Outdoor-Hour-Data-Feb-2020_MECH_VENT_5X.csv (mechanical ventilation data for winter 2020) - IC-Meter-QR-Indoor-Outdoor-Hour-Data-Feb-2020_NOTECH_5Y.csv (NOTECH ventilation data for winter 2020) Data are available under the terms of the Creative Commons Attribution 4.0 International license (CC-BY 4.0).
