# Indoor environmental quality in schools: NOTECH solution vs. standard solution

**DOI:** 10.12688/f1000research.130633.3

**Published:** 2024-04-12

**Authors:** Carlo Volf, Klaus Martiny, Mathias Andersen, Bodil Engberg Pallesen

**Affiliations:** 1 NID-Group, Copenhagen University Hospital, Copenhagen, Frederiksberg, 2000, Denmark; 2 DTI, Danish Technological Institute, Aarhus, 8000, Denmark

**Keywords:** Natural ventilation – indoor environmental quality (IEQ) – schools – mechanical ventilation – energy performance

## Abstract

**Background:**

In many Danish schools, the indoor environmental quality (IEQ) is challenged and studies document a poor IEQ in a majority of existing schools. Municipalities cannot afford comprehensive renovations and expensive mechanical ventilation solutions, hence public schools often suffer from poor indoor environment conditions. This study tests a new façade based, demand-controlled ventilation solution called NOTECH in the renovation of school. The study tests NOTECH vs. existing mechanical ventilation solution, comparing performance of both solutions at Skovbrynet Skole in Denmark.

**Methods:**

The project investigates the effect of the NOTECH solution in a primary school classroom, comparing it to a similar classroom with conventional, mechanical ventilation. Methodically, indoor environmental quality and energy performance is monitored in the two identical classrooms during one school year 2018 - 2019.

**Results:**

The results show that both systems keep the conditions within acceptable limits and CO2 levels below 1000 ppm, which is the requirement according to the Danish Building Regulations. In terms of costs, the NOTECH system has a lower overall cost than the mechanical ventilation system, with total estimated costs for installation, heating, electricity and maintenance amounting to approximately 35% of the mechanical system’s costs. Finally, the results show that the NOTECH solution has a smaller embedded CO2 footprint for building materials, reducing the estimated carbon load by 95% compared to the mechanical ventilation solution.

**Conclusions:**

While the performance of the both systems complies to the Danish Building Regulations, the indoor environmental quality between systems differs significantly. Results showing a higher air-temperature and lower relative air-humidity in the classroom with mechanical ventilation during winter and lower CO _2_ levels in the mechanically ventilated classroom during winter and summer. Costs for implementation, energy consumption for heating and CO _2_ footprint for building materials are significantly lower for the NOTECH solution, compared to the mechanical solution.

## Introduction

Historically, natural ventilation has played a crucial role in the architectural planning of schools, providing an acceptable indoor environmental quality, both during the summer and during the winter period. Often, a good, balanced indoor climate was established in a very low-tech manner; pupils typically spending 45 minutes in each lesson, in high-ceilinged classrooms, often followed by 15 minutes of venting during breaks, in empty classrooms and with children outdoor in the school yard year-round. This meant that in most cases, natural ventilation - which most schools were fully based on until the mid1960s – probably did meet the requirement of CO _2_ < 1.000 ppm, based on natural air-intake from windows and doors. ^1^ Recent studies document that natural air-intake from windows and doors remains a functional and sustainable solution for schools. ^2^^,^^3^ Yet, today the planning of schools is quite different, almost entirely based on mechanical ventilation. The building codes increasingly demanding mechanical ventilation. ^4^^,^^5^ However, also the situation and teaching philosophy is different today. First of all, schoolchildren spend more time indoors. According to a report from Organization for Economic Cooperation and Development, OECD (2016), ^6^ Danish pupils spend a total of 10,960 hours on school education. This is considerably more time than the European average of 7,540 hours. Secondly, lessons tend to have a longer duration today, the children often having 2-period lessons corresponding to 1.5 hours. Thirdly, the architecture of the schools has changed. Together with energy renovation and air-tight mechanically ventilated buildings, new types of classrooms have been introduced, such as project space schools and open-plan schools. Older schools however, built before 1995, still have traditional classrooms based on natural ventilation through the windows. These schools make up 90% of all schools in Denmark according to a Danish study from 2016 by Toftum and Wargocki. ^7^ According to the study, more than half of these existing schools in Denmark (1,300) suffer from a poor indoor air quality. Only 40-44% of Danish schools had a “good” indoor air quality (CO _2_ below 1.000 ppm). This indicates that traditional, natural ventilation through windows and doors may not be sufficient anymore.

**Figure 1.**
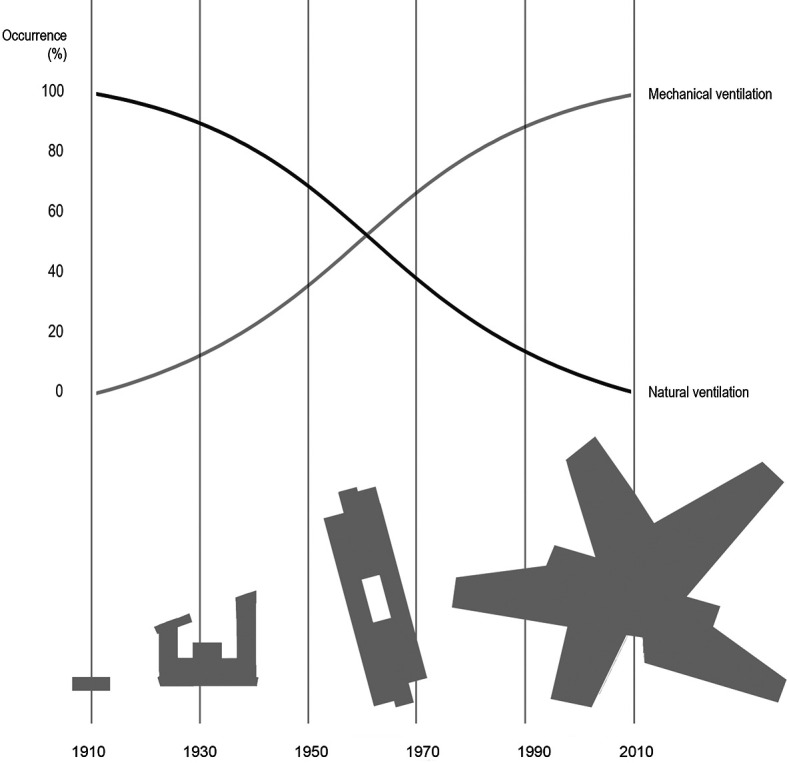
Illustrating a simplified development in typical Danish school typologies over the last 100 years in the period 1910 to 2010, and an estimated corresponding development in ventilation principles (top), changing from fully natural to fully mechanical ventilation. From the village school (1910) to the aula school and an early example of the open plan school, up to the project school.

A few years later, another study from 2022 ^8^ investigating 234 Danish schools and 709 classrooms confirmed this and found that 53% of the classes had higher CO _2_-concentration levels than the allowed threshold of 1000 ppm. This study showed largely the same result as the earlier study from 2016, despite a number of initiatives to correct the inadequate ventilation. Even though slightly more classes than before had a ventilation system, the air quality did not improve significantly. Based on measurements in 60 Danish schools, with a total of 245 classrooms, the researchers found that ventilation had a positive effect on sick leave among teachers. The study also showed that generally increased air change per hour resulted in students’ performance improving by 9%. Furthermore, lowering the temperature from 25 °C to 21 °C resulted in an 8% improvement in student performance. However, 91% of 245 classrooms in Denmark exceeded the recommended upper limit of 1.000 ppm CO _2_ in some periods during the school hours. On average, the 1.000 ppm CO _2_ limit was exceeded during 47% of the school hours during the heating season (November – April) and 12% during the summer season (May - October). The results also indicated that general noise levels were too high (above 65 dB(A)) in the classrooms in 63% of the usage time.

These findings are supported by other studies by e.g. Heschong Mahone Group (1999), ^9^ Tanner (2009) ^10^ and Shendell *et al*., 2004, ^11^ which suggested that a poor indoor climate often resulted in more noise in the classroom, reduced concentration ability and poorer learning. Better air quality and better light quality also have shown to increase students' concentration and performance in mathematics and reading, among other subjects (Barrett *et al.,* 2015 ^12^ and Grün and Susanne Urlaub, 2015). ^13^ A study (Kjeldsen *et al.* 2015) ^14^ suggested a link between indoor climate parameters, such as room temperature (°C), CO _2_ levels (ppm), daylight (lux), relative humidity (%) noise level (dB(A)), and performance, with these parameters having an effect on the well-being, learning and general health of students.

Economically, a Danish study in 2018 ^15^ showed that maintaining a good indoor climate costs approximately 0.38-0.54 EUR per pupil per day, using conventional, mechanical ventilation, corresponding to approximately 2.100 – 3.000 EUR per class annually. This represents a heavy burden for schools and municipalities, and may be the reason why the renovation of schools is happening slowly in Denmark. Approximately 50% of Danish schools were built in the 1960s and 1970s, and so many schools need renovations. Only approximately 10% of all schools have been built according to the energy requirements in the Building Regulations 1995 and onwards, and only a fraction of these, approximately 5%, are built according to the energy requirements for buildings from 2006. ^8^ In other words, more than half of the Danish schools face an urgent need for energy renovation and improvement of the indoor climate in order to meet the current requirements.

## Methods

**Figure 2.**
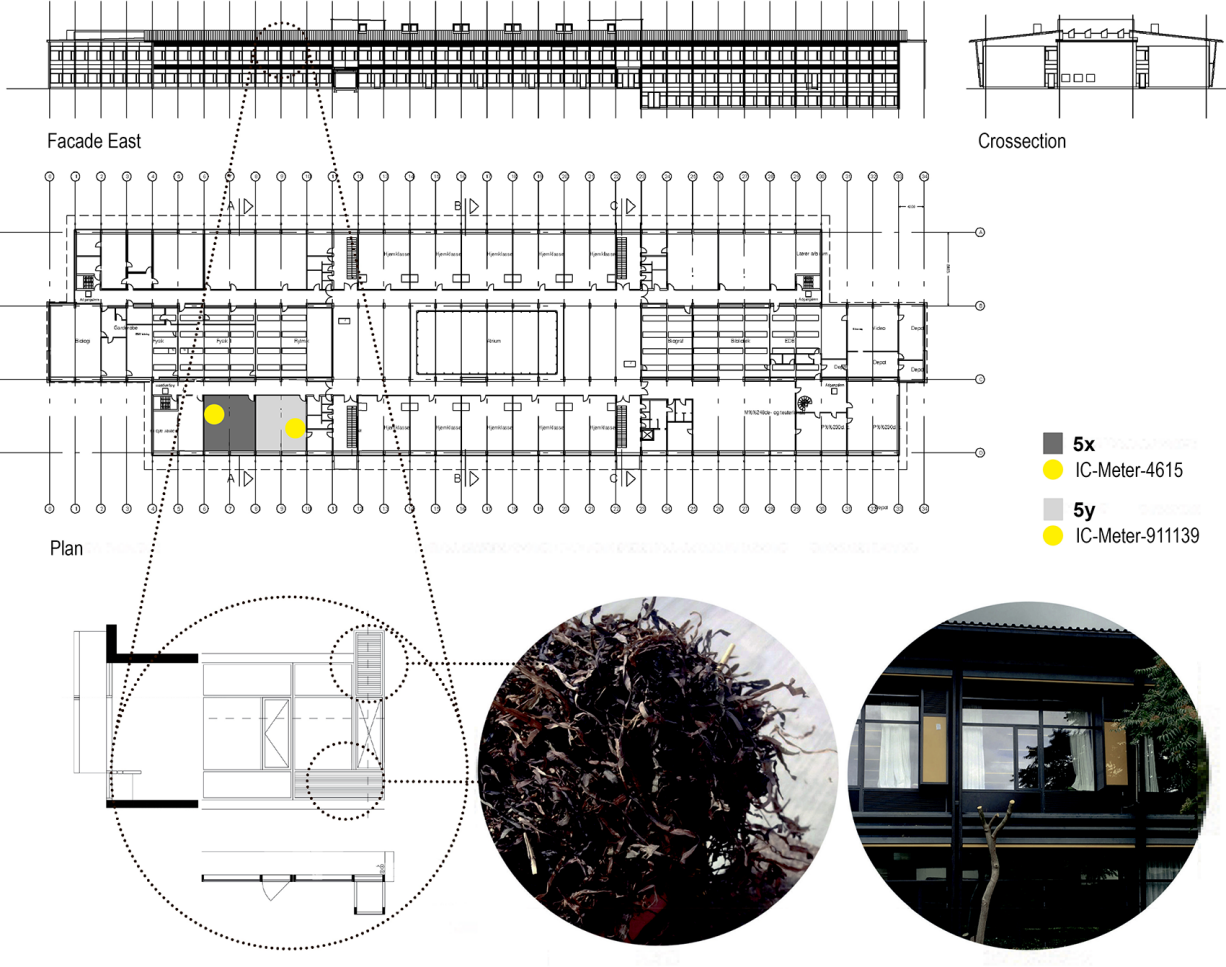
Plan, East-facing façade and cross section showing classroom with mechanical ventilation (5x, dark grey) and classroom with Notech ventilation (5y, light grey). The indoor environmental quality was measured 1.8 m above floor level on wall, suing IC-Meter-911139. SBi-17, Box ID: 2DF19FEE. In classroom 5y, the Indoor environmental quality was measured 1.8 m above floor level on wall, using IC-Meter-4615. SBi-27, Box ID: 19FCA309.

Methodically, the study monitored the performance of the NOTECH solution and compared it to a traditional mechanical ventilation (MechVent) solution. The systems were implemented in identical, east-facing classrooms at Skovbrynet Skole in Copenhagen. See Figure 2. The school was built in 1968 and renovated in 2022. The school was chosen as a representative, typical Danish school, since a majority of the Danish schools are built in the 1960s. Originally, all these schools were based on natural ventilation through façade windows, however today they are often being renovated to fully mechanical ventilated schools.

**Figure 3.**
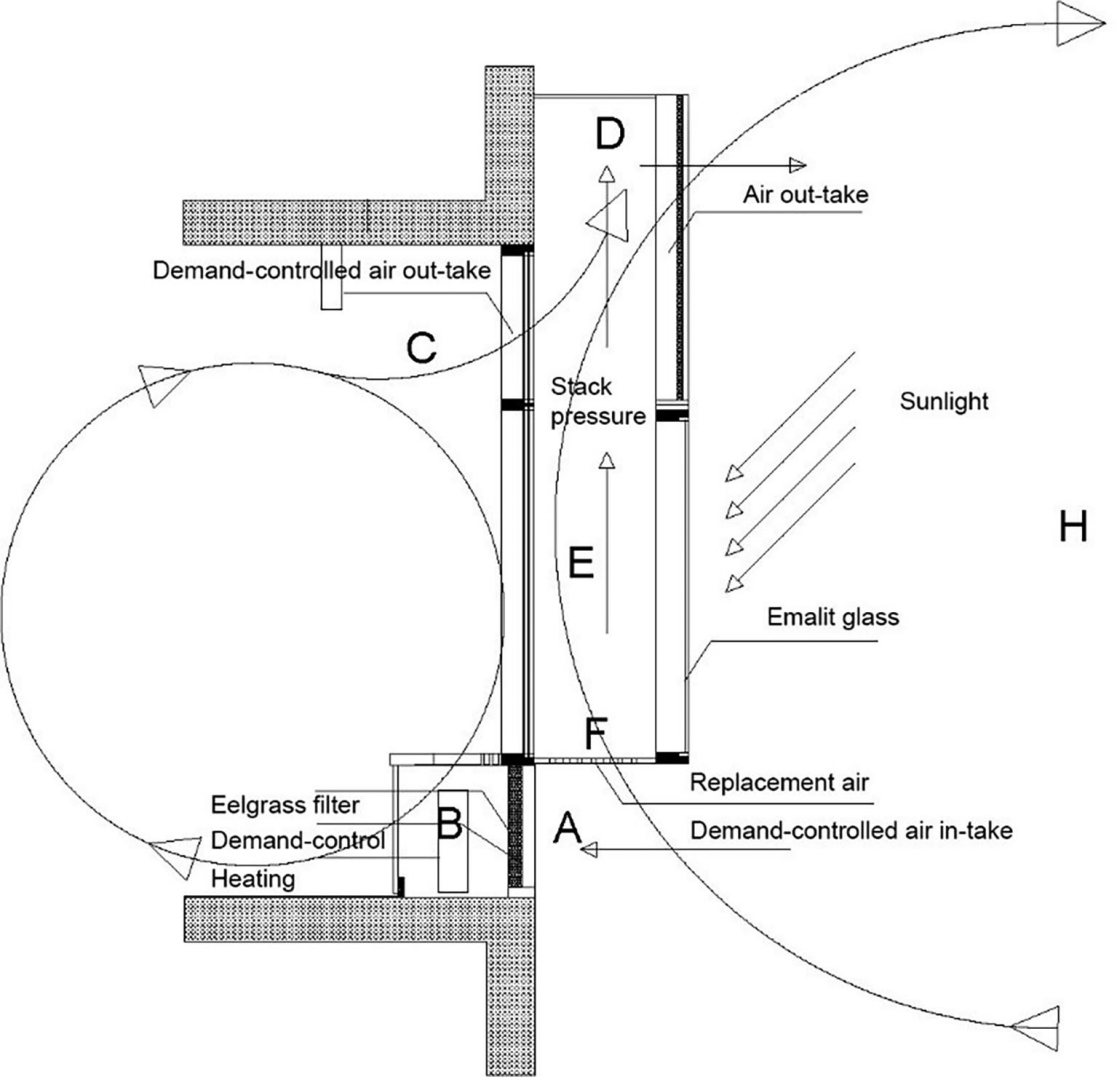
The NOTECH system, vertical cross section of façade. Top: A solar chimney (E, D) provides under-pressure and IoT demand-controlled dampers (A) control fresh air-intake at the same time filtering the fresh air through special designed eelgrass filters (B). Bottom: Each classroom has two solar chimneys outside with air outlet above ceiling height.

The aim of this study was to see, if it was possible to use demand-controlled natural ventilation instead when renovating schools from this period. Inclusion criteria for the classrooms were set to 5 ^th^ grade pupils representing the median of a normal 9-10 years period of schooling. The NOTECH system included façade elements, windows, blinded windows, solar chimneys for supplementary air outlet, and louvres for air intake with eelgrass filtering of outdoor air. Intelligent demand-controlled outlets on the top of the classroom and intakes at the bottom of the classroom were continuously adjusting the NOTECH ventilation rates according to temperature and CO _2_ levels inside as well as weather conditions outside. NOTECH was based on passive one-sided ventilation design, combined with stack pressure, since the inlet was placed low, preheated and then due to stack pressure passed to the outlets; see Figure 3. In comparison, the mechanical ventilation system was an active system, providing a constant air-change throughout the usage time, with conventional air-tight construction and conventional materials such as mineral wool, steel and wood.

### Description of classrooms

Two classrooms were included in the study, both dimensioned for 24-28 pupils, each having a total of 16 pupils and 1-2 teachers. Both classrooms measured 9.5 x 8.6 m, with a net area of 81 m ^2^. The classroom with the mechanical ventilation system had recessed ceiling with a ceiling height of 2.7 m. The classroom with the NOTECH system had a total ceiling height of 3.2 m, with no recessed ceiling, since NOTECH was a façade-based ventilation system. Thus, the volumes were 220 m ^3^ for the classroom with the mechanical system and 262 m ^3^ for the test room with the NOTECH system.

The two classrooms had identical materials, structure and furniture, except for the façades. In the classroom with the NOTECH system, clear glazing (g = 0.72) were introduced, while the reference classroom was based on standard glazing (g = 0.56). The total glass-area of the classroom with the NOTECH solution was 14 m ^2^ (glass-floor area ratio of 17.3%) whereas the total glass area of the MechVent solution was 16.1 m ^2^ (glass-floor area ratio of 19.9%). Both classrooms had exterior yellow solar shadings for adjustable solar protection and two openable windows.

### Description of the systems

The ventilation principles of the two classrooms were fundamentally different. The NOTECH system was a passive ventilation system, driven by thermal stack pressure, while the system in the reference classroom was driven by a central, mechanical ventilation unit. Both systems fulfilled the minimum requirements of 5 l/s/pupil and 0.35 l/s/m ^2^ floor area. The mechanical ventilation in the reference classroom was similar to that of the rest of the school.

*The MechVent solution*

The mechanical ventilation system was based on an existing standard unit serving a number of other classrooms, driven by motors running at an installed power of 2x4 kilo-Watts and demand-controlled by a clock timer in the IBS system. Via a (IBS) timer, the system was set automatically to start ventilating the classroom at 7 am, one hour before the start of teaching and end at 5 pm. The capacity of the standard mechanical ventilation system was dimensioned for a maximum airflow of approximately 500 m ^3^/h per classroom. During the experiment, the airflow was estimated to be running at 80% of the full capacity. The mechanical system had a SFP value of 1.8 kW/m ^3^/s and a heat recovery value (HRV) of 0.8.

*The NOTECH solution*

The demand-controlled NOTECH system was controlled and tuned based on the number of pupils. The total area of two outlets was 0.7 m ^2^, while the air intake was 2.1 m ^2^, approximately three times larger than the outlet area (0.7 m ^2^), allowing lower airflow speed at high air change per hour (ACH), without the risk of draught. We measured the air-flow on selected days at noon using an anemometer. The capacity of the NOTECH system was dimensioned to a maximum airflow of approximately 765 m ^3^/h per classroom. The capacity with fully opened outlets and intakes was based on max. air velocity in the outlets. This was measured to 0.3 m/s, corresponding to 765 m ^3^/h, meeting the requirements of 33 m ^3^/h. The NOTECH system was set to automatically ventilate from 07.30 am and end at 14.30 pm, since the demand-controlled system only ventilated during the usage hours. The demand-control of the NOTECH system was tuned to an estimated airflow of 50% of full capacity, based on the number of pupils. The tuning was regulated twice, on October 6 ^th^ 2019 from 20 to 22°C and again the Dec 20 ^th^ to 21.5°C. The later adjustments were based on a wish to optimize and balance the ventilation rate, at the same time making sure that no pupils nor teachers found the room too chilly. Adjustments only affected the setpoint of temperature, not the setpoint of timer.

Beside from natural ventilation, the NOTECH system integrated two elements 1) clear glazing and 2) natural materials, as described below.

*Clear glazing*

The system introduced high-transmittance windows based on two-layered low-iron clear glass that provided more transmitted daylight in the form of UV, visible and IR light. ^16^ The window allowed a clearer view out. It also allowed parts of UVB light to penetrate, which may have positive health effects such as enabling vitamin D formation, ^17^ as well as germicidal effects and killing of bacteria/viruses. ^18^ During winter periods, the NOTECH solution was designed to utilize to a higher degree solar light and heat, using high transmitting, clear glazing, with a higher g-value, thus facilitating a higher passive solar heat gain in the heating season.

*Natural materials*

The NOTECH solution was based on eelgrass, since it enables air to pass through with only a small transmission loss in the filter of approximately 5 Pa, enabling efficient air-flow using natural ventilation, at the same time reducing risk of high draught rates and outside noise. Eelgrass is a vernacular material, resistant to bacteria and weathering, etc due to the natural salinity. Therefore, the material traditionally has been used in mattresses for hygienic reasons. Eelgrass is also known to have large biophilic surface, together with its natural salt and mineral content, this enables it to easily absorb and release humidity. Furthermore, the eelgrass material do not release unwanted particles. This was tested in VOC-studies in 2018, ^19^ were eelgrass achieved cradle2cradle platinum certification on material health. Natural materials such as eelgrass are known to have a antimicrobial effects, because of its natural salt-content and antimicrobial secondary metabolites. ^20^ When applied in larger quantities, these natural materials help maintain a higher, more natural, relative humidity, being a hygroscopic building material, absorbing and releasing moist. In this way, eelgrass helps balance the humidity, maintaining a relative humidity 40% - 60% RH, which is a recommended minimum threshold value for a healthy environment. ^21^^,^^22^

### Monitoring procedure and time plan

Data were simultaneously collected for both classrooms (NOTECH and standard mechanical ventilation) using two identical Indoor Climate meters (IC-Meter Mobile (GSM), in each classroom, placed 1.8 m above the floor in each classroom. See Figure 2. IC-Meters logged indoor climate parameters CO _2_, temperature and relative humidity every 5 minutes. Data were collected continuously over one year and analyzed during the summer and winter, respectively. Typical periods were chosen as 3-week periods during the warmest and coldest periods. Respectively with temperatures above 20 C during summer and temperatures below 10 C during winter, in order to represent a normal Danish summer and winter setting. See Table 1.

**Table 1.** Time plan 2019-2020 showing baseline measurements and effect measurements. Baseline measures were not included in the results and only served as a baseline for the parallel health study to confirm that the two classrooms were identical before intervention.

PERIOD	Baseline	Measurement dates	Representative weeks
Summer period (off heating season)	08.03.19. – 15.03.19	15.08.19 – 21.10.19	26.08.19 – 15.09.19
Winter period (heating season)		22.10.20 – 11.03.20	17.02.20 – 11.03.20

### Description of data collection

*Indoor environmental quality*

Hourly average values of CO _2_ concentration, relative humidity and temperature were analyzed for the representative periods, as averages, cumulated frequency of different CO _2_ levels, relative humidity (% RH) and temperature. Data on the two glass types used in the study document a higher transmittance of the NOTECH system’s glazing types (g = 0.72, visible light transmittance = 0.82) providing more transmitted daylight and solar heat compared to the mechanical system (g = 0.6, visible light transmittance = 0.74). Differences in daylight between the classrooms were not measured, since the focus of the study was on CO _2_ levels, relative humidity (% RH) and temperature levels.

*Energy performance*

The operation costs for electricity of both systems, excluding energy costs for lighting, were calculated as EUR per pupil per month in total estimated electricity consumption, based on a Danish national kWh price of 0.30 EUR (2019/2020), with an estimated average of 20 children in each classroom, 7 h per day, and the standard usage period of 25.2 days per month (corresponding to 200 days per year).

Operation costs of both systems were based on data from the Public Municipality of Gladsaxe, serving as an external, independent evaluator. Data were based on on-site measurements, using digital Ista Oprimo III evaporation meters. A digital evaporation meter functioning by updating the energy consumption, quite comparable to mercury evaporators, indicating the relative consumption of heating by a number. The evaporation meters were installed directly on the convector heaters in each classroom 0.5 m above floor level. Installation was carried out at the same day and evaporation meters were monitored every 15 minutes during the heating season, providing a separate and comparable data between the systems. At the end of the study, this number was collected by third parties and correlated to the total energy consumption of the school (kWh). Based on the gross heated area of 11,080 m ^2^, and the size of each classrooms (81 m ^2^), the specific, annual energy consumption for heating as well as costs for heating were calculated for each classrooms.

*Estimated total installation costs*

In order to represent a realistic school setting, the calculations of energy performance for electricity and heating, together with the estimated installation costs, were based on a total of 10 classrooms for both systems. This corresponded to the capacity of the existing mechanical ventilation system for each section in the school. Each section consisting of two identical floors, each having a separate mechanical ventilation system. See Figure 2. All operating costs for electricity, heating and maintenance together with installation costs were based on a 20-year lifetime, since this represents a normal full lifespan of a mechanical ventilation system.

*Carbon footprint*

The approximated life-cycle analysis (LCA) was based on the European ECO _2_ carbon footprint for materials and products with a focus on wooden building products. ^23^ Material calculation for the mechanical system was based on data and description of materials from the suppliers. ^24^ Material calculation for the mechanical system was based on one central, mechanical system, Danvent Combi Aggregate TC, with heat recovery running on 240 V, with galvanized steel ducts. Material calculation for the NOTECH system was based on 2 pcs IoT-controlled chain actuators (WMX 804) in steel and 4 pcs Klimatek dampers in aluminum, running on 24 V Belimo motors.

*Parameters for intelligent demand-control*

The NOTECH system was demand-controlled by three parameters: usage time, temperature levels and CO _2_ levels, taking into account the number of pupils. The standard mechanical system was controlled by one parameter: usage time, not taking into account the number of pupils in the classroom. Occupancy data were not included in the study, since it would be complicated and since both classrooms had comparable number of children and teachers during the entire study.

*Analysis*

Indoor environmental quality parameters for a 3-week representative period during summer and winter, were represented in grouped in intervals in pie charts showing a percentage of the different ranges observed in order to compare the two systems across seasons. In the study, both classrooms had identical occupation scheme. We did not collect occupancy data for the classrooms. All data on indoor environmental quality were analyzed in a t-test.

## Results

### Indoor environmental quality

The overall results of the indoor environmental quality showed the following significant differences between the two ventilation systems.

**Figure 4.**
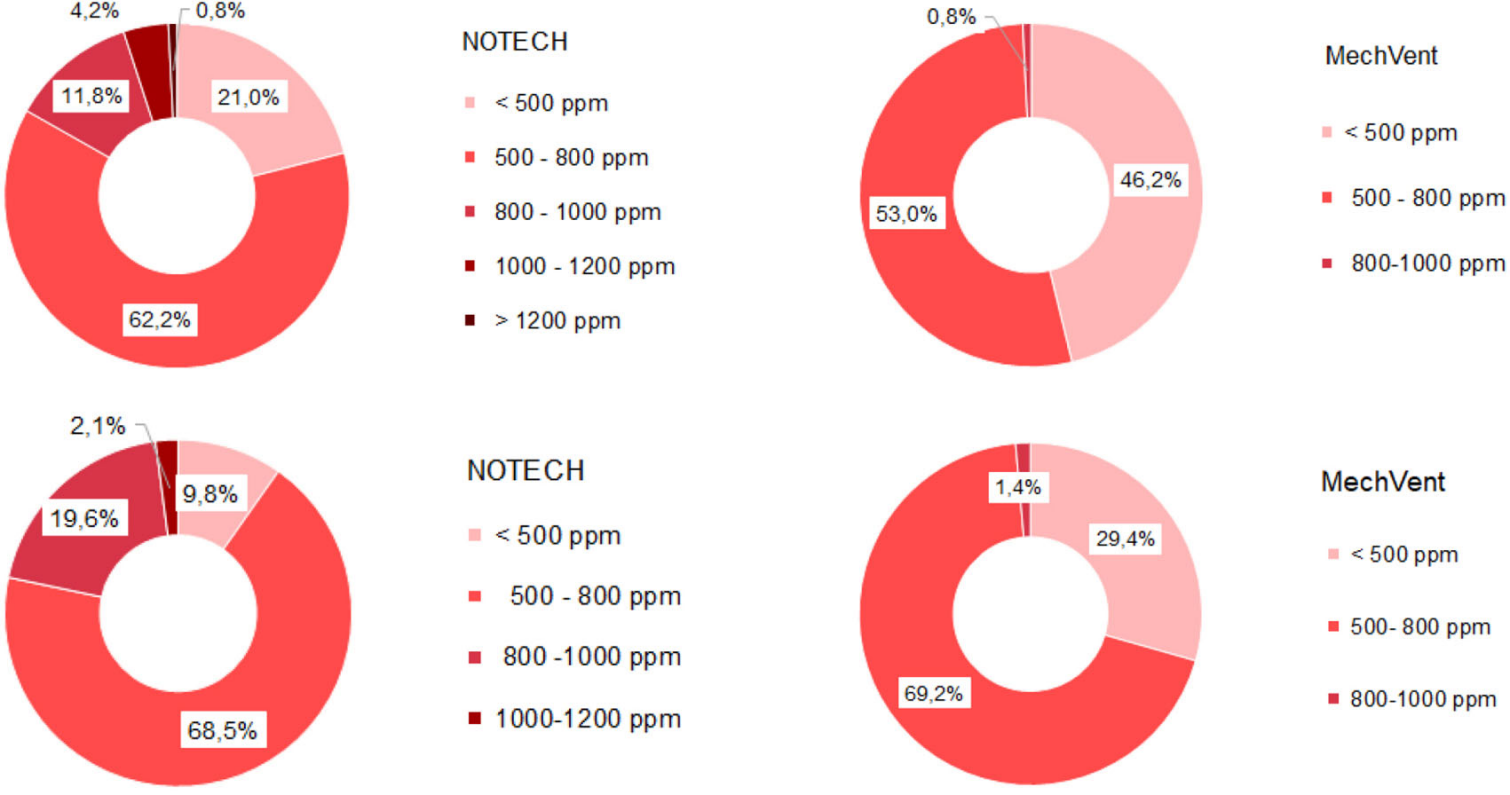
Frequency (%) of indoor classroom CO _2_ levels (without correction for outdoor CO _2_ levels). NOTECH and mechanical ventilation summer (above) and winter (below).

**Figure 5.**
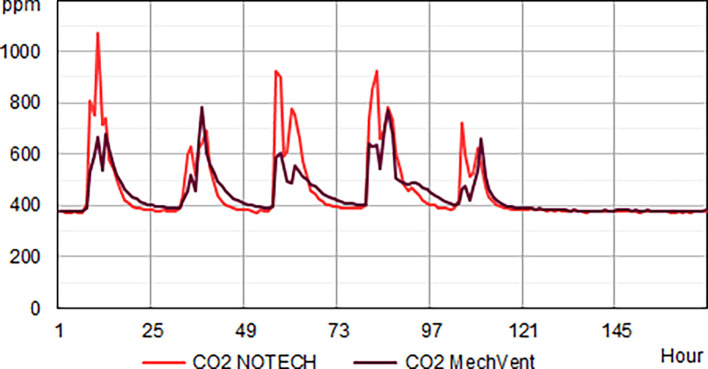
CO _2_ levels for one winter week. Typical average hourly levels for NOTECH natural ventilation compared to mechanical ventilation (MechVent).

Indoor classroom CO _2_ levels were lower than 1.000 ppm for 100 % of school hours for the mechanical system and 95% of the school hours for the NOTECH system in the summer period 2019 and winter period 2020 ( Figure 4). CO _2_ levels above 1.000 ppm were reached for the NOTECH system in short periods of 10-15 minutes, 5% of the usage time, during the summer period ( Figure 4). CO _2_ levels for the mechanical system generally were significantly lower than for the NOTECH system during winter (p=1,021E-08) as well as during summer (p=2,135E-10) when analyzed in t-test ( Figure 5). On average during the summer and winter period, the CO _2_ levels were 600 ppm for the mechanical vs. 800 ppm for the NOTECH system. Higher CO _2_ levels were reached during the summer period than during the winter period for the NOTECH.

**Figure 6.**
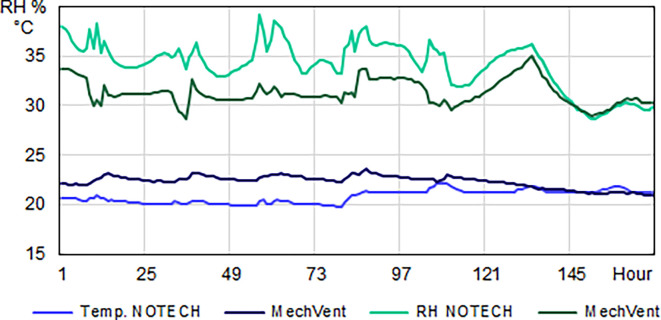
Temperature (°C) and relative humidity (%) for one winter week. Typical hourly average levels for NOTECH natural ventilation compared to mechanical ventilation (MechVent). Because NOTECH uses cold “fresh” outside air, the indoor temperature is lower (20 °C) and the relative humidity (%) higher compared to mechanical ventilation.

**Figure 7.**
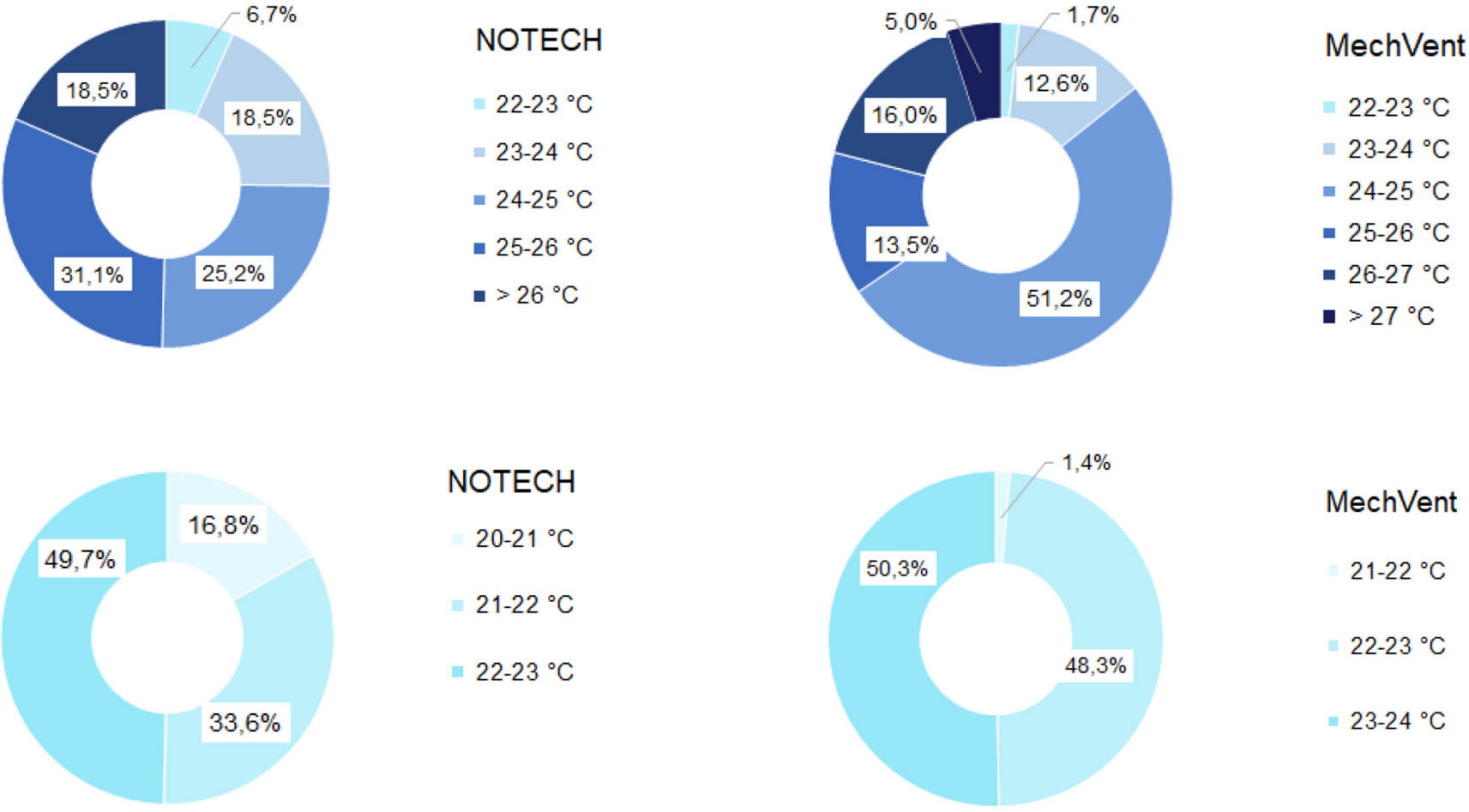
Frequency of temperature (% **)** for NOTECH and mechanical ventilation in summer (above) and winter (below).

The indoor temperature for the NOTECH system was typically about 2-2.5°C lower than the temperature for the mechanical system (MechVent) ( Figure 6). NOTECH used outside air directly without preheating, hence control of the indoor temperature (> 20°C) was a determining factor for the ventilation rates. During the summer period, no significant differences between systems were observed. The NOTECH system, provided comfortable temperatures of 20-24°C 25.2% of the time, with no hours with temperatures above 27°C. While the mechanical system provided comfort temperatures of 20-24°C 15.3% of the time during summer, and the hours with temperatures above 27°C added up to 5% of the usage time ( Figure 7).

The high-transmittance glazing types (g = 0.72) in the NOTECH system did not cause more hours with temperatures above 27°C during the summer period. Temperatures above 26°C were observed 18.5% of the time for the NOTECH system, compared to 21% of the time for the mechanical system, Figure 7.

Temperatures during winter showed significant differences. Generally air temperatures were colder for the NOTECH system, with the lowest temperatures recorded between 20 and 21°C, observed 16.8% of the usage time, whereas no temperatures below 21°C were observed in the classroom with the mechanical system (p=1,566E-22) in t-test.

**Figure 8.**
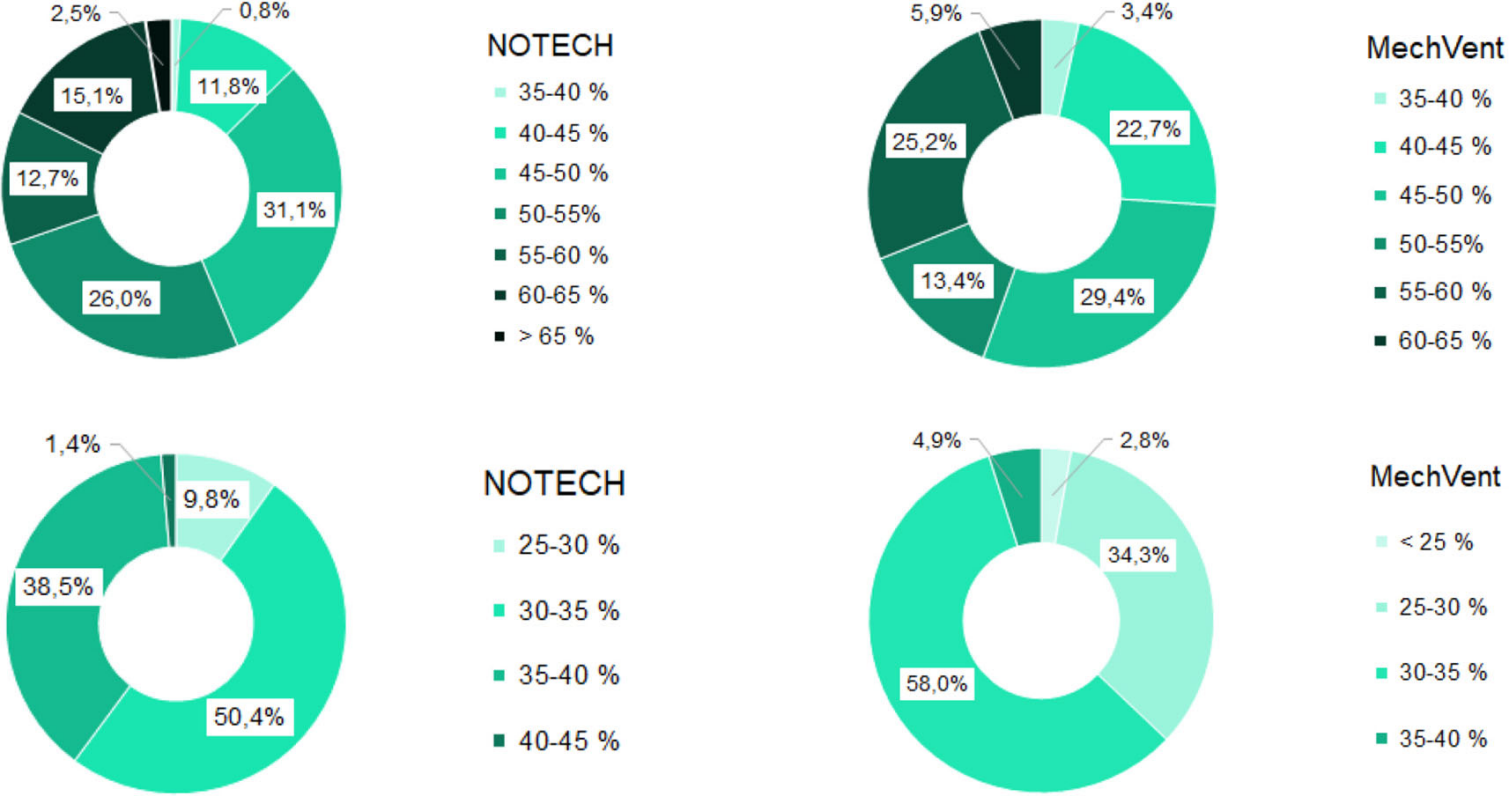
Frequency (%) of relative humidity For NOTECH and mechanical ventilation in summer (above) and winter (below).

During the summer period, the relative humidity was roughly the same for both systems, see Figure 8. This is despite the fact that the temperatures generally were higher for the classroom with the mechanical system. During the winter period, the classroom with mechanical ventilation had a significantly lower relative humidity, compared to the NOTECH system (p=1,717E-15). The relative humidity for the NOTECH system was between 35-45% RH 39.9% of the time, while a relative humidity between 35-45% was observed only 4.9% of the time for the mechanical system. The general indoor winter temperatures generally were higher for the mechanical system during winter, thus lowering the relative humidity; see Figure 8.

### Energy cost

The energy costs of electricity for both systems were estimated based on a total of 10 classrooms with 20 pupils 7 h per day, 25.2 days per month and kWh price set at 0.30 EUR. The estimated annual running cost for electricity was based on a 20 year lifetime. Estimated annual electricity consumption per classroom for the standard mechanical system was 305 kWh, amounting to 91 EUR annually per classroom. Estimated annual electricity consumption per classroom for the NOTECH system was 7 kWh, amounting to 2 EUR annually per classroom.

**Figure 9.**
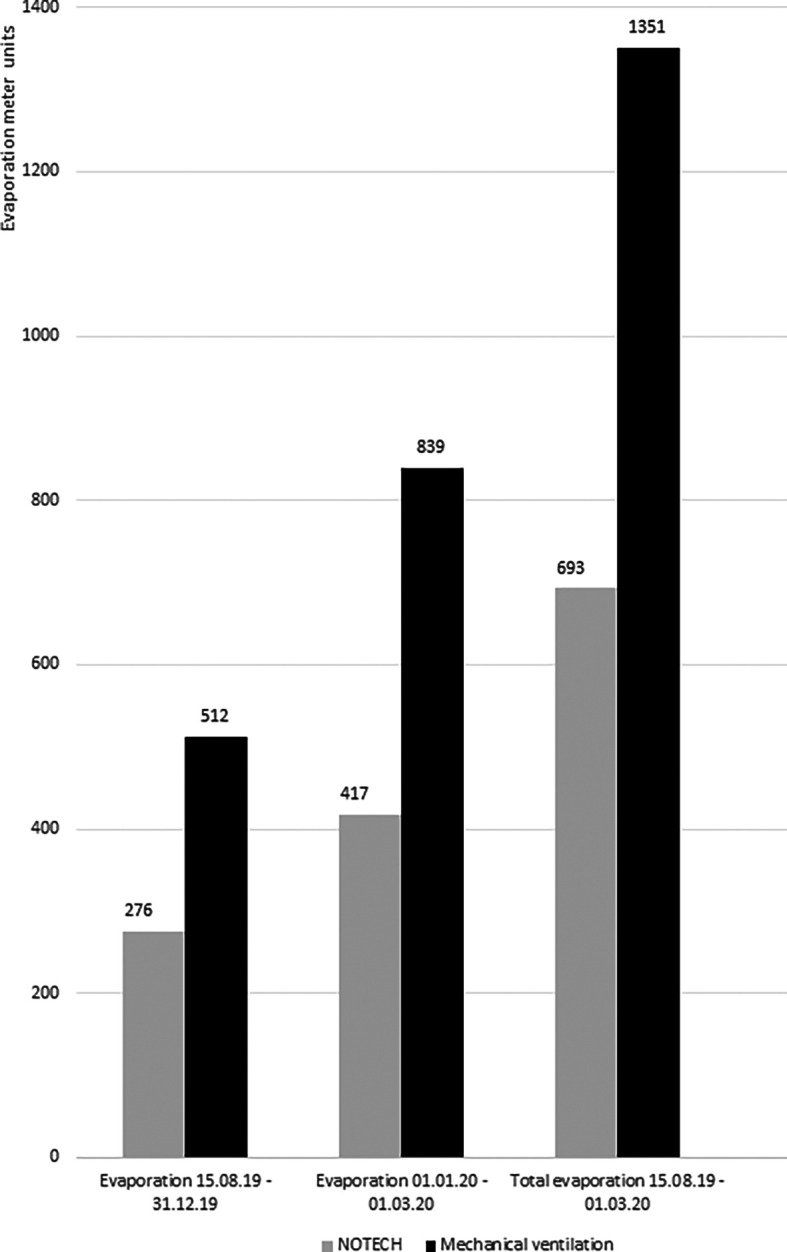
Evaporation meter registrations for NOTECH system vs. mechanical ventilation. The monitored data from evaporation meters monitoring each system during two periods. The first period is 15.08.2019-31.12.2019 and second period is 01.01.2020-01.03.2020. Source: Ista-meter 839, 891, 952, 072.

Energy consumption for heating was measured in the classrooms for NOTECH and the mechanical system, respectively. The energy for heating during the heating season (October-March), as measured by Ista evaporation meters, showed cumulated 693 units for the NOTECH system and 1,351 units for the mechanical system. Monitoring was carried out in two periods, the first period was for 2019 (15.08.2019-31.12.2019) and second period was for 2020 (01.01.2020-01.03.2020) ( Figure 9).

Based on third part data, collected by Gladsaxe Municipality, the degree day corrected total energy consumption for Skovbrynet Skole for heating October 2019 – April 2020 was 1,358 MWh for a gross heated area of 11,080 m ^2^, giving the following average energy consumption for the two systems based on 81 m ^2^ classrooms and 0.30 EUR per kWh. Based on Ista measurements, the energy consumption for the heating season 2019/2020 was 62,9 kWh/m ^2^ per year for the NOTECH system and122,6 kWh/m ^2^ per year for the mechanical system. Costs for heating per classroom in the heating season 2019/2020 amounted to1.540 EUR per year for the NOTECH system and 3.000 EUR per year for the mechanical system, based on 0.3 EUR/kWh.

### Installation cost

The estimated total installation costs were calculated based on data from Skovbrynet Skole, based on information from suppliers of the NOTECH system (WindowMaster Ltd and Velfac Ltd) and the mechanical system (DanVent Ltd). All installation costs were based on 10 classrooms, each having 20 children for both systems and a lifespan of 20 years. The total installation costs for the NOTECH system were all in all 48.450 EUR and the total installation costs for the mechanical system were 147.800 EUR (40.300 EUR for the ventilation system and 107.500 EUR for ducts, fixtures, filters, etc.).

### Total estimated costs

The total estimated costs for energy, installation, heating and maintenance per day per pupil (based on 20 pupils per classroom and 200 school days per year) was 1.06 EUR for the NOTECH system and 3.00 EUR for the conventional mechanical system. All in all, the total annual estimated costs of the NOTECH system amounted to 35% of the conventional, mechanical system; see Table 2.

**Table 2.** Total annual estimated costs for energy, installation, heating and maintenance (every half year based on an estimated, average 20 year lifetime).

	Electricity	Heating	Installation	Maintenance	Total
	EUR/classroom per year	EUR/classroom per year	EUR per year	EUR/classroom per year	EUR/classroom per year
NOTECH	2	1.540	2.400	300	4.242
MechVent	91	3.000	7.650	1,250	11.991

### CO _2_ in building materials

**Figure 10.**
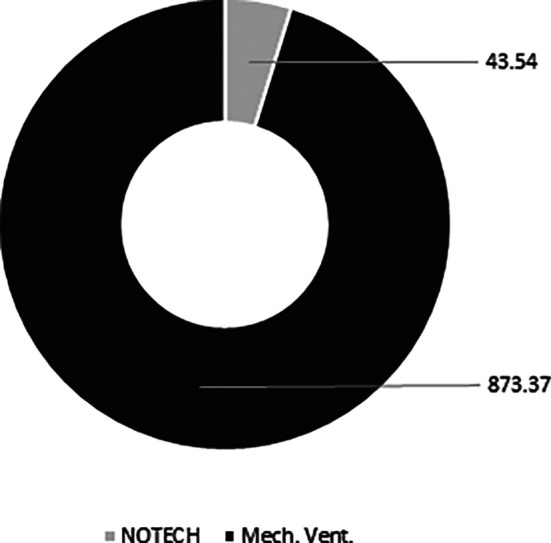
Estimated overall carbon footprint (kg CO _2_) for NOTECH system vs. mechanical system based on 10 classrooms. Embedded CO _2_ in the building materials is estimated for a standard life time period of 30 years. ECO _2_ data for materials used is standard values from Ruuska. ^18^

The NOTECH solution was based on natural, bio-based materials, thus the CO _2_ footprint was smaller. The conventional mechanical solution spent 873 kg CO _2_, while the NOTECH solution spent 44 kg CO _2_. Thus, the NOTECH system reduced the embedded CO _2_ in the building materials by a total of 95%, compared to the mechanical ventilation system ( Figure 10).

## Discussion

The aim of this study was to make a comparison between the NOTECH system and a conventional mechanical ventilation system. The classrooms in the study were representative for schools from the 1960s and 1970s and the results of this pilot study therefore should be seen as “a proof of concept” for this type of schools. The two rooms were alike though differing in volume due to the recessed ceiling height (0.5 m) in the classroom with the mechanical ventilation system – a difference which is considered representative for the solutions, since NOTECH as a façade solution, is not taking up ceiling space.

### Indoor environmental quality

The airflow in the mechanical system was controlled by a clock-timer, while the NOTECH system was demand-controlled by three parameters: usage time, temperature levels and CO _2_ levels. Except for short periods, both systems could keep the CO _2_ levels and temperatures within acceptable limits under the given conditions in the actual measurement periods. The usage of the rooms were comparable, each hosting 16 Fifth grade pupils.

*CO _2_ levels*

Generally, higher values of CO _2_, temperature and humidity tended to follow a higher activity and person load in the classrooms. Lower CO _2_ levels were generally observed during the summer period, compared to higher levels in the winter period. For both systems the summer period showed increased hours with low CO _2_ levels (below 500 ppm), indicating that the pupils were spending more time outdoor during summer – since inside activity at these periods would have shown an increase in the CO _2_ levels (above 500 ppm). However, higher CO _2_ levels were reached during the summer in the NOTECH solution. The natural, temperature gradient driven ventilation in NOTECH did not provide a constant air change per hour. The demand-control of the NOTECH system was tuned and slightly adjusted during the winter period, to provide a slightly higher air change during the winter period. We cannot tell if these adjustments caused lower CO _2_ levels in general during winter. Windows could be manually opened in both classrooms and other unknown factors were present. Normally the winter period is “the critical” period for natural ventilation systems, however, the demand-control of the NOTECH system in this pilot study seems to meet the requirements, providing sufficient fresh air in 95% of the usage time at a lower cost, during both the summer and winter periods being tested. However, it should be kept in mind that the winter 2019/2020 was mild compared to a normal Danish winter.

*Temperature*

The results show a lower number of hours with temperature above 26°C for the NOTECH system during the summer period, compared to the mechanical system (18.5% for NOTECH compared to 21% for mechanical ventilation) ( Figure 7). The fact that the glass area in the NOTECH system was reduced by 13% compared to the standard mechanical system may explain these results. The higher frequency of hours with temperatures above 26 °C (21%) in the standard mechanical system may also be explained by the fact that the NOTECH system provides generally lower room temperatures, passively cooling during the morning and daytime. Difference in air volumes in the classrooms may also have influenced the temperatures. Increased ceiling height and thus greater air volume acts as a buffer, reducing excessive temperatures. This may be another explanation for the higher frequency of temperatures above 26 °C in the classroom with the mechanical system, compared to the classroom with the NOTECH system. Taken together, these factors may all add up to explain the differences between the two systems.

*Relative humidity*

During the winter period, the relative humidity generally tended to be higher for the NOTECH system. The lower limit <30% RH was reached more frequently for the mechanical system (37.1%) compared to the NOTECH system (9.8%) ( Figure 8). This partly was caused by the general lower temperature in the classroom with natural ventilation. The heat recovery of the mechanical ventilation system together with differences in airflow – the NOTECH system being laminar and the mechanical system being turbulent airflow – may have had an influence on this, but the effects were not examined in detail in this study. In addition to a single-sided focus on set limits of CO _2_ concentrations, these results may suggest to include other parameters for a more satisfying indoor environmental quality, e.g. a better relative humidity.

### Energy

This study reveals several differences between the two systems when it comes to energy performance and sustainability. The NOTECH system in general provides a sufficient air-change-per-hour at a lower estimated total annual cost, with estimated annual costs being 65% less compared to the mechanical system.

This study found that NOTECH saved energy for heating: 59.7 kWh/m ^2^ per year compared to the standard system, corresponding to a reduction of 51% for heating. This was not expected, since the NOTECH system being a passive system taking in cold, outside air directly without preheating, while the mechanical system having a heat recovery value (HRV) of 0.8. However, the energy consumption for heating is based on complex and dynamic factors (such as solar heat gain, weather/wind/outdoor and indoor temperatures, etc.). The function of the NOTECH system needs to be investigated further, especially during cold winter periods. Generally, the NOTECH system had a lower air change per hour, which may explain the lower energy consumption for heating compared to the mechanical system. The energy consumption for heating fluctuates from year to year and data in this study were collected empirically during one year and through a mild winter period. A cold winter period may result in colder indoor air-temperatures or in a higher general energy consumption for heating, and remains to be examined further during cold winter periods.

### Costs

Our estimated installation and running costs for the mechanically ventilated indoor climate system in this pilot study are based on 10 classrooms and 20 years lifetime, the installation cost for the NOTECH system is estimated to save 66%, compared to the conventional, mechanical ventilation system. The difference between the two systems in the estimated total capital expenditure (operating costs for electricity, heating, maintenance and installation costs) shows that the NOTECH system has a markedly lower cost compared to the mechanical system, costing only 35% of a mechanical ventilation system in total running costs. Based on a classroom with 20 pupils, the estimated total costs for the NOTECH are 1.06 EUR per day per pupil, while the costs for the mechanical ventilation system are 3.00 EUR per day per pupil.

### Life cycle assessment

The approximated LCA analysis, based on the European ECO _2_ carbon footprint for building data for materials and products with focus on wooden building products, showed that the NOTECH system had a far smaller CO _2_ footprint, being only 5% of the CO _2_ footprint of the mechanical system (see Figure 10). In this respect, NOTECH is better in line with future sustainability requirements such as e.g. Level(s) in the European Commission and Denmark’s New Class of Sustainable Architecture. ^25^ According to a recent study, ^26^ waste and materials from the building industry today make up 50-83% of the emitted CO _2_ over a period of 80 years. Today, mechanical ventilation represents a heavy CO _2_ footprint often not fully accounted for. ^27^ Making an effort to reduce the carbon footprint of the building materials by 95% is a significant and valuable contribution to a more sustainable architecture and an important result of this pilot study of the NOTECH system.

### Limitations of study

The limitations of the study first of, lies in the fact that it compares two fundamentally different systems. This makes it difficult to make any exact comparison between the systems. The small sample size of this study, also is a limitation. For practical reasons, we only implemented and studied two different systems in two different classrooms. Both classrooms were identical in size, patterns of occupancy and age of children, justifying the small sample size. However, we did not collect occupancy data during the study and do not know the exact number of children. A total of 16 pupils and 1-2 teachers in both classes is lower than average in Danish school classes, which is 24 pupils. The demand-controlled NOTECH system compensated for this, while the classroom with mechanical system did not. The usage time between systems also differed, the mechanical system ventilating for a 2.5 hour more than the NOTECH system. When analyzing data for three-week periods during summer and during winter, we assumed that the number of children were comparable between classrooms. Data on indoor environmental quality were collected only for representative 3-week periods. These periods represent the coldest and the warmest periods over the one-year study period, respectively. Thus they are considered to represent and cover any annual variations. The results of this study only represents Skovbrynet Skole and results should be viewed only in the context of Skovbrynet Skole. However, when this is said, Skovbrynet Skole represents a majority of Danish school constructed during the 1960s. Hence, results of this study might be useful to several other schools. More research, and studies with larger sample sizes, are needed to uncover different indoor climate strategies as well as the means, by which to improve the indoor climate. Especially, taken into consideration, that a majority of Danish schools from this period today are facing modernization and renovation over the coming decades. In this context, natural ventilation is relevant to test and implement, since it seems that natural ventilation has potentials and benefits, when it comes to sustainable, low cost, renovation of schools.

## Conclusion

This pilot study shows a proof-of-concept for the NOTECH system in typical Danish school settings at Skovbrynet Skole. Further studies are needed to conclude that the demand-controlled system can also meet the acceptable levels under dimensioning conditions, i.e. with 24-28 students in the classrooms and during colder winter periods. The classrooms are representative for schools built in this period and the findings are representative for similar cases. Likewise, the following conclusive points based on the specific case study are:

• With the person load of the classrooms both systems could keep both the CO _2_ level and temperature within acceptable limits. CO _2_ levels for the mechanical system though, were significantly lower than for the NOTECH system during winter (p=1,021E-08) and during summer (p=2,135E-10 in t-test).
• The indoor room temperature during winter showed significant differences. Generally air temperatures were cooler for the NOTECH system, with the lowest temperatures recorded between 20 and 21°C, observed 16.8% of the usage time, whereas no temperatures below 21°C were observed in the classroom with the mechanical system (p=1,566E-22 in t-test). Having a lower set-point when compared to the mechanical system, the NOTECH system resulted in more hours with comfortable temperatures between 20-24°C, compared to the mechanical system and fewer hours of temperatures above 27°C during the summer period when compared to the mechanical system.
• During the winter period, the relative humidity was significantly higher for the NOTECH system. The classroom with mechanical ventilation had a lower relative humidity, when compared to the NOTECH system (p=1,717E-15 in t-test). The lower limit <30% RH was reached more frequently for the mechanical system (37.1%) compared to the NOTECH system (9.8%). Variations in temperature and relative humidity generally tended to follow high activity and person load in the classrooms.
• The estimated total costs for energy costs (energy consumption for electricity and heating) were lower for the NOTECH system compared to the mechanical system. The energy consumption for heating showed that the NOTECH system accounted for only 51% of the energy consumption, corresponding to 62.9 kWh/m ^2^ per year compared to 122.6 kWh/m ^2^ per year for the mechanical system, during the heating season tested (October 2019 - April 2020). Usage time between the two systems though was different. Usage time for NOTECH system was 07.30 – 14.30 versus 07.00 – 17.00 for the mechanical system. This accounts for some of the differences in energy consumption between the systems. Also, the setpoint for heating was lower for the NOTECH system (20.5 C) compared to the mechanical system (22 C).
• The total estimated installation costs for the NOTECH system amounts to 35% of the total installation costs of the conventional, mechanical system used in this study. However, the systems had a different start-time and end-time; NOTECH had a shorter operation time, 2.5 hours shorter than the mechanical system.
• Over a period of 20 years, the estimated total running costs of the NOTECH system were 65% cheaper than the estimated total running costs of the mechanical system.
• According to the life cycle assessment (LCA) over 30 years, the NOTECH system was estimated to reduce the carbon emissions of the building materials, the embedded CO _2_, by 95% compared to that of the mechanical ventilation system.

Since the NOTECH system is a passive system, primarily using energy for heating, the winter period is decisive for the total energy balance of NOTECH. In this study, the results show that NOTECH reduced the energy consumption for heating during a mild winter period – which is contrary to what could be expected during a normal Danish winter. This needs to be further investigated.

This pilot-study challenges the current building requirements when it comes to demands for mechanical ventilation. The results indicate that intelligent control of passive, natural ventilation systems can be a sustainable solution, if not fully replacing a mechanical ventilation system then supplementing mechanical systems. The NOTECH system did have lower energy consumption, less impact on the architecture of the school, used fewer materials and therefore had a lower environmental impact. The results suggest that other types of buildings, with rooms having a lower person load than classrooms, can also benefit from demand-controlled natural ventilation systems such as NOTECH.

## Ethical considerations

This study did not include any human participants.

## Data Availability

Zenodo: Indoor Environmental Quality in Schools: Notech Solution vs. Standard Solution. https://doi.org/10.5281/zenodo.7821202 (Volf, 2023). This project contains the following files:
- IC-Meter-QR-Indoor-Outdoor-Hour-Data-Aug-2019_MECH_VENT_5X.csv (mechanical ventilation data for summer 2019) - IC-Meter-QR-Indoor-Outdoor-Hour-Data-Aug-2019_NOTECH_5Y.csv (NOTECH ventilation data for summer 2019) - IC-Meter-QR-Indoor-Outdoor-Hour-Data-Feb-2020_MECH_VENT_5X.csv (mechanical ventilation data for winter 2020) - IC-Meter-QR-Indoor-Outdoor-Hour-Data-Feb-2020_NOTECH_5Y.csv (NOTECH ventilation data for winter 2020) Data are available under the terms of the Creative Commons Attribution 4.0 International license (CC-BY 4.0).
