# Supplementary material for: Indoor environmental quality in schools: NOTECH solution vs. standard solution
Source: F1000Res. 2024 Apr 12;12:560. Originally published 2023 May 30. [Version 3] doi: 10.12688/f1000research.130633.3 (PMC11016168; doi:10.12688/f1000research.130633.3)
Supplement: Peer review history

# Reviewer report: Reviewer response for version 3

**DOI:** 10.5256/f1000research.163914.r265851

**Published:** 2024-04-22

**Authors:** Alo Mikola

**Affiliations:** 1 Tallinn University of Technology, Tallinn, Estonia

I don't have any new comments.

Is the work clearly and accurately presented and does it cite the current literature?

Yes

If applicable, is the statistical analysis and its interpretation appropriate?

Yes

Are all the source data underlying the results available to ensure full reproducibility?

Partly

Is the study design appropriate and is the work technically sound?

Partly

Are the conclusions drawn adequately supported by the results?

Partly

Are sufficient details of methods and analysis provided to allow replication by others?

Partly

Reviewer Expertise:

NA

I confirm that I have read this submission and believe that I have an appropriate level of expertise to confirm that it is of an acceptable scientific standard.

# Reviewer report: Reviewer response for version 3

**DOI:** 10.5256/f1000research.163914.r265849

**Published:** 2024-04-13

**Authors:** Antonio Martinez-Molina

**Affiliations:** 1 Drexel University, Philadelphia, Pennsylvania, USA

Authors addressed all my concerns.

Is the work clearly and accurately presented and does it cite the current literature?

Partly

If applicable, is the statistical analysis and its interpretation appropriate?

Yes

Are all the source data underlying the results available to ensure full reproducibility?

Yes

Is the study design appropriate and is the work technically sound?

Partly

Are the conclusions drawn adequately supported by the results?

Yes

Are sufficient details of methods and analysis provided to allow replication by others?

Partly

Reviewer Expertise:

IAQ, HVAC, Therm Comfort, Building Controls, Natural Ventilation.

I confirm that I have read this submission and believe that I have an appropriate level of expertise to confirm that it is of an acceptable scientific standard.

# Reviewer report: Reviewer response for version 2

**DOI:** 10.5256/f1000research.156406.r231678

**Published:** 2024-01-25

**Authors:** Antonio Martinez-Molina

**Affiliations:** 1 Drexel University, Philadelphia, Pennsylvania, USA

The authors chose a very current and evolving topic; a topic that would benefit from further investigations. The paper is therefore relevant and has the potential to fit well into the current theoretical paradigm. Nevertheless, in my opinion has an important methodological problem while describing the data collection campaign. There are no relevant references regarding the data gathering approach such as air temperature, relative humidity, carbon dioxide. There is a need to reference what method/regulation has been used while monitoring each pollutant (i.e. ASHRAE, OSHA, maybe something local, etc.)These type of references need to be shown in the manuscript in order to have a reliable and trustworthy scientific investigation.

Is the work clearly and accurately presented and does it cite the current literature?

Partly

If applicable, is the statistical analysis and its interpretation appropriate?

Yes

Are all the source data underlying the results available to ensure full reproducibility?

Yes

Is the study design appropriate and is the work technically sound?

Partly

Are the conclusions drawn adequately supported by the results?

Yes

Are sufficient details of methods and analysis provided to allow replication by others?

Partly

Reviewer Expertise:

IAQ, HVAC, Therm Comfort, Building Controls, Natural Ventilation.

I confirm that I have read this submission and believe that I have an appropriate level of expertise to confirm that it is of an acceptable scientific standard, however I have significant reservations, as outlined above.

# Response

**Published:** 2024-03-14

**Authors:** Carlo Volf

**Affiliations:** Copenhagen University Hospital, Denmark

Dear Antonio Martinez-Molina,

 Thanks a lot for you comments and your feedback to our manuscript. We have now revised the ms. (version 3).

 Below, I have collected our answer on your question. Please find it there as well as in the revised manuscript.

 My Best Regards

 Carlo Volf

 Q: The authors chose a very current and evolving topic; a topic that would benefit from further investigations. The paper is therefore relevant and has the potential to fit well into the current theoretical paradigm.

 Nevertheless, in my opinion has an important methodological problem while describing the data collection campaign. There are no relevant references regarding the data gathering approach such as air temperature, relative humidity, carbon dioxide. There is a need to reference what method/regulation has been used while monitoring each pollutant (i.e. ASHRAE, OSHA, maybe something local, etc.)These type of references need to be shown in the manuscript in order to have a reliable and trustworthy scientific investigation.

 Thanks a lot for your important comment. We have added a description of the data collection campaign under method and results and added a t-test on indoor environmental quality parametres; air temperature, relative humidity and carbon dioxide. We used IC-metres to collect data on the indoor environmental quality and elaborated this in the ms. Since it was a real school setting, we could not do laboratory tests of pollutants and regulation – instead we chose identical 3-week periods (the coldest winter period vs. the warmest summer period, respectively). We did a comparative analysis of to  5 ^th^ grade classrooms - with the same occupancy- and usage pattern. Please find the revised ms. at F1000Research.com.

# Reviewer report: Reviewer response for version 2

**DOI:** 10.5256/f1000research.156406.r231675

**Published:** 2024-01-17

**Authors:** Insung Kang

**Affiliations:** 1 Department of Civil Engineering, The University of Texas at Arlington, Arlington, Texas, USA

The manuscript entitled “Indoor environmental quality in schools: NOTECH solution vs. standard solution” investigates the effects of a passive ventilation system “NOTECH” on indoor environmental quality and energy efficiency, as compared to a mechanical ventilation system in a primary school classroom. However, due to some issues of the design of the proposed system and some analysis methods, I would need to see a new version with updated study design and its results.

1. First and foremost, authors conclude that the performance of the two systems roughly is the same in relation to the indoor environmental quality, temperature, CO2 levels and relative air-humidity. However, there seems to be significant differences in CO2 levels as well as T/RH between two systems, as 600 ppm vs 800 ppm. This should be statistically demonstrated that the differences in those parameters are not significant, e.g., t-test or Mann Whitney test. Additionally, sensitivity analysis for seasonality if further needed.
2. Also, authors mention that the design of NOTECH is chiefly passive, one-sided thermal ventilation relying on buoyancy effect. However, in one-sided ventilation strategy, the amount of air that can be exchanged through a single opening is generally less than what can be achieved with wind-driven natural ventilation in cross ventilation rooms. Therefore, the measurement ventilation rates of both NOTECH and mechanical ventilation system should be added and explicitly mentioned as a result.
3. What is the filtration efficiency of the eelgrass filters and how much they block the air flow?
4. Authors collected heating costs as an operation cost for comparing two ventilation systems. However, considering comparing two ventilation strategies, why heating costs were considered? Are they additional heating load due to the amount of ventilation air? If yes, why cooling costs were not considered?
5. Following that, during the winter season, additional energy needed for adding heat to the natural ventilation air should be generally higher than mechanical ventilation system, which has heat recovery ventilator (HRV)?
6. Regardless, the total annual estimated costs for energy, installation, heating, and maintenance in Table 2 need accuracy check and cross validated by the external evaluator(s).  

Is the work clearly and accurately presented and does it cite the current literature?

Partly

If applicable, is the statistical analysis and its interpretation appropriate?

No

Are all the source data underlying the results available to ensure full reproducibility?

Yes

Is the study design appropriate and is the work technically sound?

No

Are the conclusions drawn adequately supported by the results?

No

Are sufficient details of methods and analysis provided to allow replication by others?

Partly

Reviewer Expertise:

Architectural Engineering, Built Environment, HVAC Systems, Indoor Air Quality, Building Simulations

I confirm that I have read this submission and believe that I have an appropriate level of expertise to state that I do not consider it to be of an acceptable scientific standard, for reasons outlined above.

# Response

**Published:** 2024-03-14

**Authors:** Carlo Volf

**Affiliations:** Copenhagen University Hospital, Denmark

Dear Insung Kang,

 Thank you very much for your important  and valuable input to our manuscript. We have now revised the ms.

 In the following I have added our answers to your questions as well as a short description of the revisions made to the ms.

 My Best Regards

 Carlo Volf

 Q: First and foremost, authors conclude that the performance of the two systems roughly is the same in relation to the indoor environmental quality, temperature, CO2 levels and relative air-humidity. However, there seems to be significant differences in CO2 levels as well as T/RH between two systems, as 600 ppm vs 800 ppm. This should be statistically demonstrated that the differences in those parameters are not significant, e.g., t-test or Mann Whitney test. Additionally, sensitivity analysis for seasonality if further needed.

 Thanks a lot for this comment. Both systems though kept levels within demands in the Danish Building Requirement. We have changed this passage – we have specified that the performance of both systems met the building codes and the requirements. We have specified the differences. They are both significant as we also mention in the revised version of the paper.

 We have also performed at T-test. CO2 Results show that there is a significant difference between the systems in summer: p = 0.0000000002135 and in winter: p = 0.000000001021. Temperature: Results showed significant difference during winter: p = 0,0000000000000000000001566. %RH: Results showed significant difference during winter: p = 0,000000000000001717.

 Data 17/2-11/3 2020 and 26/8-15/9 2019.

                                            T-test CO2    T-test Temp  T-test RH%                                              

 P-value, Summer               2,135E-10      0,176             0,657                                                        

 P-value, Winter                  1,021E-08      1,566E-22      1,717E-15                                                

 P-value, Summer+Winter  1,970E-17      0,005             0,109                                                        

 Q: Also, authors mention that the design of NOTECH is chiefly passive, one-sided thermal ventilation relying on buoyancy effect. However, in one-sided ventilation strategy, the amount of air that can be exchanged through a single opening is generally less than what can be achieved with wind-driven natural ventilation in cross ventilation rooms. Therefore, the measurement ventilation rates of both NOTECH and mechanical ventilation system should be added and explicitly mentioned as a result.

 Thanks a lot for this comment. Notech is based on passive one-sided ventilation design, combined with stack pressure, since the inlet is placed low, preheated and then due to stack pressure passed to outlets. We do not use wind-driven natural ventilation at schools, since classrooms often have closed doors to adjacent rooms/facades. We measured the air change – using an anemometer. The total area of two outlets 0.7 m2 had a max. air velocity of 0.3 m/s. This corresponds to 765 m3/h. We only measured the air-flow on selected days at noon with fully opened outlets and intakes. We have described and elaborated this in the revised manuscript.

 Q: What is the filtration efficiency of the eelgrass filters and how much they block the air flow?

 Thanks a lot for this comment. The transmission loss in the filter is approximately 5 Pa providing both airflow, reduced draught and reduced outside noise. We have described and elaborated this in the revised manuscript.

 Q: Authors collected heating costs as an operation cost for comparing two ventilation systems. However, considering comparing two ventilation strategies, why heating costs were considered? Are they additional heating load due to the amount of ventilation air? If yes, why cooling costs were not considered?

 All operating costs for electricity, heating and maintenance together with installation costs were based on a 20-year lifetime. This lifespan was chosen, since it represents a normal full lifespan of a mechanical ventilation system. We wanted to see if natural ventilation was more expensive since it caused more need for heating – as often reported. We did not find this to be the case though. We did not add any additional heating load. The additional heating load for the mechvent can be a result of a constant ACH in mechvent – it also can be the result of general higher room temperatures – we have described this in the discussion part and elaborated this further and a little more precise in the revised manuscript. In Denmark at 56th N latitude we do not plan cooling in existing (school) buildings. We added costs to make an economical comparative analysis based on the case study.

 Q: Following that, during the winter season, additional energy needed for adding heat to the natural ventilation air should be generally higher than mechanical ventilation system, which has heat recovery ventilator (HRV)?

 We thought that Notech would result in adding heating compared to mechvent. However we were surprised this was not the case. Perhaps because the Notech system is an intelligent, demand controlled ventilation system – which enables it to reduce ventilation (and hence demand for heating) when possible. The mechvent system did have a HRV = 0.8 however it did not have demand control and was a standard existing mechanical ventilation system in existing schools. This makes a difference when it comes to additional energy needed. Also the usage time was a little shorter for the Notech system, as mentioned in the paper. We have elaborated this a little further in the revised manuscript.

 Q: Regardless, the total annual estimated costs for energy, installation, heating, and maintenance in Table 2 need accuracy check and cross validated by the external evaluator(s). 

 We have based this estimate Based on third part data, collected by the Public Municipalities – serving as an external, independent evaluator. We have elaborated this and changed the overview to an estimated overview. Please see the revised manuscript.

 Again, thank you so much for your valuable feedback and important comments to the manuscript, please find revised ms.( version 3) at F1000Reserch.com.

 .

# Reviewer report: Reviewer response for version 2

**DOI:** 10.5256/f1000research.156406.r211358

**Published:** 2023-10-13

**Authors:** Alo Mikola

**Affiliations:** 1 Tallinn University of Technology, Tallinn, Estonia

At the same  time, I still do not understand the working principle of used „evaporation meters“.

 In article it is said that „Ista Oprimo III evaporation meters“ were used. I searched the internet and found such a device:

https://www.ista.com/dk/infocenter/doprimo-iii-radio-info-folder/

 First thing is that this is not „Ista Oprimo III“, it is „Ista **D**oprimo III“. Maybe it's not the device, that was actually used. Second thing is that I really do not understand why this devide is colled evaporation meter. Isi t really something like this: https://www.google.com/search?client=firefox-b-d&q=evaportaiopn+meter#vhid=3WQusN_ydipbDM&vssid=3981:xstATSSLGqS5qM

 If possible, this topic could be specified in the article.

Is the work clearly and accurately presented and does it cite the current literature?

Yes

If applicable, is the statistical analysis and its interpretation appropriate?

Yes

Are all the source data underlying the results available to ensure full reproducibility?

Partly

Is the study design appropriate and is the work technically sound?

Partly

Are the conclusions drawn adequately supported by the results?

Partly

Are sufficient details of methods and analysis provided to allow replication by others?

Partly

Reviewer Expertise:

NA

I confirm that I have read this submission and believe that I have an appropriate level of expertise to confirm that it is of an acceptable scientific standard.

# Response

**Published:** 2024-03-14

**Authors:** Carlo Volf

**Affiliations:** Copenhagen University Hospital, Denmark

Dear Alo Mikola,

 Thanks a lot for your review of our ms. To shortly answer your questions, the Istametres we used were implemented by third parties and mounted at the heaters - third party was the municipalities in Gladsaxe. The devices measured the heating indirectly by evaporation (measured digitally, not using mercury). The Istametres were installed at the same date, measuring the energy consumption through one entire heating season. This subsequently was calculated by third parties; the Skovbrynet School.

 We have now revised and elaborated the ms. Please find it at F1000research.com.

 Again, thanks a lot for your input and for reviewing our manuscript.

 My Best Regards

 Carlo Volf

# Reviewer report: Reviewer response for version 1

**DOI:** 10.5256/f1000research.143403.r189714

**Published:** 2023-08-15

**Authors:** Alo Mikola

**Affiliations:** 1 Tallinn University of Technology, Tallinn, Estonia

The topic of this manuscript is novel and the paper has a practical value to give better overview of indoor air quality and ventilation systems in case of school buildings. The topic is particularly important in light of the spread of the coronavirus. At the same time, I have some questions and recommendations that should be taken into account before publishing the article.

- It is hard to understand the methods and results of the measurements of sound pressure levels of studied ventilation systems. Is this possible to add more detailed description what are the required noise levels in Danish classrooms, how exactly the measurements were conducted, what was the measuring period in both case, were people in the classroom during the measurement period, which measuring instruments were used, what was the background noise level during the measuring period.
- The required noise level should be compared to the measurements results. At the moment there are just the results brought out.
- Are the results of sound pressure level (corrected with A-filter) described in paper. If so, please use the correct terminology.
- If the sound pressure level is above the permissible level for both systems how could such systems be accepted by the customer?
- If the noise level where measured during the during the period of use, then the analyze do not show the noise from ventilation system and I'm not sure if the noise level should be analyzed in the article in such a case.
- The description of the noise levels in paper should be added closer to the Figure 8. If the Figure 8 is far away from the analysis chapter, it is hard to read the paper.
- Can You describe what kind of measurements where done using “evaporation meter”, what is the main working principle of this device?
- Page 7 – “Operation costs of both systems were based on measurements using digital Ista Oprimo III evaporation meters.” This statement needs a detailed description how exactly the operational costs can be analysed according to the evaporation meter?
- The heat recovery value of studied mechanical ventilation system is not described in paper.
- The SFP values of fans of studied mechanical ventilation system is not described in paper.
- It is hard to estimate the energy calculations, if these vales are not described.

Is the work clearly and accurately presented and does it cite the current literature?

Yes

If applicable, is the statistical analysis and its interpretation appropriate?

Yes

Are all the source data underlying the results available to ensure full reproducibility?

Partly

Is the study design appropriate and is the work technically sound?

Partly

Are the conclusions drawn adequately supported by the results?

Partly

Are sufficient details of methods and analysis provided to allow replication by others?

Partly

Reviewer Expertise:

Hvac technology, energy efficiency of buildings, indoor climate of buildings

I confirm that I have read this submission and believe that I have an appropriate level of expertise to state that I do not consider it to be of an acceptable scientific standard, for reasons outlined above.

# Response

**Published:** 2023-09-19

**Authors:** Carlo Volf

**Affiliations:** Copenhagen University Hospital, Denmark

We have now added valuable informations to the article, answering your questions and adressing your concerns. For your information, we have decided not to add data on noise levels, since it is not a primary outcome of the study. Like daylight, it is a very important element. However, for the ventilation we have chosen to focus only on air-quality and temperature. In this way we think, that the findings are more precise - especially since we did not find any significant differences in noise levels between the systems. We will upload shortly.

 Thanks a lot for your valuable feedback and comments.

# Reviewer report: Reviewer response for version 1

**DOI:** 10.5256/f1000research.143403.r189717

**Published:** 2023-07-25

**Authors:** Asit Kumar Mishra

**Affiliations:** 1 School of Public Health, University College Cork, Cork, County Cork, Ireland

The work investigates a noel passive ventilation system for classrooms and compares it with mechanical ventilation system.

 As expected, the passive system consumes less energy but it also provides similar indoor conditions and has lowers embedded carbon. These are good points in favour of the new system. 

 More information needs to be provided on the monitors used for indoor environment measurements and where they were positioned. Also, it would be greatly appreciated to have images and floor plans of the classrooms studied. 

 Literature discussed needs to cover more such studies and studies that have investigated similar systems for improving ventilation, using passive and/or low energy measures.

 Comments to authors:

- Abstract - Results - "keeps within acceptable limits" - acceptable limits of what?
- Aim for shorter sentences. A lot of sentences are getting long winded and losing their track.
- Introduction - what is the meaning of balanced indoor climate?
- Reference missing for pre-1995 schools making up 90% of all Danish schools
- School construction year - there is some discrepancy, sometimes authors use 1968 and sometimes 1967
- Why specifically was eelgrass chosen for filters? This justification seems to be missing
- Equipment specs for indoor environmental monitors is missing
- Occupancy data for either classroom seems to be missing. There is value in energy cost estimates. But is this the occupancy though the entire study duration?
- Cost estimates - why do a 10 classroom 20 year estimate - some justification would be appreciated?
- A section covering study limitations would be useful for this work. An important limitation as I see it is authors compare NOTECH to mechanical ventilation. And this is understood that there are logistic limitations. But, ideally we would want a comparison against the building as it is and maybe even include some existing passive ventilation systems to compare to the novel ventilation system.
  - Along the same lines, existing literature on similar passive measures for classrooms (ones involving solar chimneys, façade modifications for comfort and air quality control) should have been discussed more.

Is the work clearly and accurately presented and does it cite the current literature?

Partly

If applicable, is the statistical analysis and its interpretation appropriate?

Not applicable

Are all the source data underlying the results available to ensure full reproducibility?

Yes

Is the study design appropriate and is the work technically sound?

Yes

Are the conclusions drawn adequately supported by the results?

Yes

Are sufficient details of methods and analysis provided to allow replication by others?

Partly

Reviewer Expertise:

Indoor climate in low energy buildings, thermal comfort standards, HVAC

I confirm that I have read this submission and believe that I have an appropriate level of expertise to state that I do not consider it to be of an acceptable scientific standard, for reasons outlined above.

# Response

**Published:** 2023-09-19

**Authors:** Carlo Volf

**Affiliations:** Copenhagen University Hospital, Denmark

We have now added valuable informations to the article, also specifying what you have asked for. We will upload it shortly. Thanks a lot for you valuable input to the article.

# Response

**Published:** 2024-03-14

**Authors:** Carlo Volf

**Affiliations:** Copenhagen University Hospital, Denmark

Dear Asit Kumar Mishra,

 Thanks a lot for you comments - I have revised the ms. according to your valluable input. Please find changes described below. A revised version 3 of the manuscript will be uploaded shortly in F1000Research..

 My Best Regards

 Carlo Volf

 We have added drawings:

Link to Figure xx

 Figure xx. In classroom 5x with mechanical ventilation (dark grey), indoor environmental quality was measured 1.6 m above floor level on wall, suing IC-Meter-911139. SBi-17, Box ID: 2DF19FEE. In classroom 5y with Notech ventilation (light grey). Indoor environmental quality also was measured 1.6 m above floor level on wall, using IC-Meter-4615. SBi-27, Box ID: 19FCA309.

 We have added more literature/references. We have elaborated your questions below and in the ms. 

 Q: Abstract - Results - "keeps within acceptable limits" - acceptable limits of what? 

 Acceptable limits according to National Building Regulations, BR18, stipulating that sufficient fresh air should be provided, so that CO² levels may not exceed 1.000 PPM.

 Q: School construction year - there is some discrepancy, sometimes authors use 1968 and sometimes 1967 

 The Skovbrynet School was constructed in 1968

 Q: Why specifically was eelgrass chosen for filters? This justification seems to be missing 

 Eelgrass was chosen since it is a vernacular material, originally known for being resistant to bacteria and weathering, etc. The material has been used traditionally in mattresses, etc. for hygienic reasons. Eelgrass also is known to have large biophilic surface with a natural salt content, absorbing and releasing humidity and hereby filtering the particles in the air. Finally the transmission loss is approximately 5 Pa, making it ideal for natural ventilation, since it is reducing risk of high draught rates, at the same time enabling an efficient airflow pr. hour.

 Q: Equipment specs for indoor environmental monitors is missing 

 In the study we used IC-Meters, see spacs in Figure xx and https://www.ic-meter.com/uk/

 Q: Occupancy data for either classroom seems to be missing. There is value in energy cost estimates. But is this the occupancy though the entire study duration? 

 In the study, we did not collect occupancy data for each classroom, however, both classrooms had identical occupation scheme with a total of 24 children and 1-2 teachers. In both classrooms, the children were Fifth grade, corresponding to 11 – 12 years of age.

 Q: Cost estimates - why do a 10 classroom 20 year estimate - some justification would be appreciated? 

 In the study, we chose a 20 year cost for maintenance and running costs.

 This estimate was chosen, since this represents a standard full lifespan of a mechanical ventilation system. In the cost estimates, in all 10 classrooms were chosen since this corresponded to the planned capacity of each section of the existing mechanical ventilation system. Each section consisted of two identical halfs, each having their own separate mechanical ventilation system. See plan drawing, Figure xx.

 Q: A section covering study limitations would be useful for this work. An important limitation as I see it is authors compare NOTECH to mechanical ventilation. And this is understood that there are logistic limitations. But, ideally we would want a comparison against the building as it is and maybe even include some existing passive ventilation systems to compare to the novel ventilation system. 

 Thanks a lot for this comment. I agree. We have added limitations of the study

   

 .
